# Application of Machine Learning in Predicting the Properties of Two-Dimensional Semiconductor Materials

**DOI:** 10.3390/nano16110650

**Published:** 2026-05-22

**Authors:** Jia Yang, Lingli Tang, Yunlong Wang, Jie Wen, Wenyuan Chen

**Affiliations:** 1 School of Electrical Engineering and Automation, Tianjin University of Technology, Tianjin 300384, China; yangjia1992@email.tjut.edu.cn (J.Y.); tllbestluckyrich@stud.tjut.edu.cn (L.T.); 20234862@stud.tjut.edu.cn (Y.W.); 2 Tianjin Key Laboratory of New Energy Power Conversion, Transmission and Intelligent Control, Tianjin University of Technology, Tianjin 300384, China; 3 Sanying MotionControl Instruments Ltd., Tianjin 300384, China; jwen@symc-tec.com; 4 Tianjin Key Laboratory for Advanced Mechatronic System Design and Intelligent Control, School of Mechanical Engineering, Tianjin University of Technology, Tianjin 300384, China; 5 National Demonstration Center for Experimental Mechanical and Electrical Engineering Education, Tianjin University of Technology, Tianjin 300384, China

**Keywords:** 2D semiconductor materials, machine learning, materials property prediction, bandgap, magnetism, density functional theory

## Abstract

The rapid evolution of next-generation electronics urgently demands high-performance functional materials. Two-dimensional (2D) semiconductors, characterized by tunable bandgaps, magnetic properties, and excellent optical and electronic properties, hold significant potential for applications in nanoelectronic devices, magnetic storage, and optoelectronics. However, the high computational cost of traditional Density Functional Theory (DFT) severely restricts large-scale high-throughput screening. Meanwhile, problems such as insufficient datasets and non-uniform data quality remain prevalent. Against this background, machine learning (ML), which captures intricate nonlinear correlations and accelerates the discovery of novel materials, has emerged as an efficient technical approach. This review systematically summarizes recent advances in ML-driven property prediction for 2D semiconductors. It first elaborates the fundamental properties and classifications of 2D semiconductors, and then compares traditional computational simulations with ML algorithms, clarifying the distinct advantages of data-driven approaches. Subsequently, this work focuses on the latest progress in predicting critical properties, including bandgap, magnetism, and other physical characteristics. For bandgap prediction, classical algorithms such as random forests are compared with deep learning models represented by graph neural networks. The results demonstrate that deep learning performs much better in low-data regimes and complex material systems. For magnetic property prediction, the impact of feature engineering strategies on model accuracy and efficiency is systematically analyzed. In addition, the research progress of other physical property prediction tasks is briefly summarized. Finally, future research directions for machine learning, including standardized materials databases, physics-informed machine learning, multimodal modeling, and the integration of machine learning with experimental and theoretical methods, are outlined to address challenges in data quality, model interpretability, and cross-system generalization ability. This work aims to provide a systematic theoretical foundation and methodological guidance for research on two-dimensional semiconductor materials assisted by machine learning.

## 1. Introduction

Since the first successful exfoliation of graphene in 2004, two-dimensional (2D) material systems have rapidly become an important part of research in materials science. Beyond graphene, extensive experimental and theoretical studies on various emerging two-dimensional layered materials, including silicene, blue phosphorus, transition metal dichalcogenides (TMDs), and MXenes, have further demonstrated the controllable fabrication and tunable structural properties of these 2D materials on different substrates [1]. Among them, 2D semiconductor materials have demonstrated great application potential in fields such as electronics, optoelectronics, energy, and catalysis, owing to their unique physical and chemical properties. For instance, 2D semiconductor materials such as WS_2_ and MoS_2_ play a significant role in the development of future electronic components and are regarded as promising alternatives to silicon crystals, making low-cost mass production and application of certain advanced technologies possible in the future. In the research on 2D semiconductors, variations in key properties critically determine their performance in practical applications. Accurate and rapid prediction of material properties can help researchers gain deeper insights into material behavior, thereby facilitating the development and innovative application of new materials and related devices. Consequently, the prediction of key properties has become an essential aspect of 2D semiconductor research, with significant implications for advancing the field.

In the property prediction of 2D semiconductor materials, traditional approaches involve synthesis, characterization, property testing, and application exploration of the materials. These approaches involve the use of scanning electron microscopes [2], physical property testing [3], first principles calculations [4], and molecular dynamics simulations [5]. However, these methods, which rely on extensive repetitive trial-and-error experiments and high-precision calculations based on first principles, are time-consuming, laborious, and expensive. Therefore, finding a rapid, accurate, and economical solution is a priority for this research domain. In recent years, with the development of electronic information technology, machine learning (ML), as an essential branch of artificial intelligence (AI), has achieved remarkable success in various fields such as healthcare [6], autonomous driving [7], and energy management [8] by collecting and analyzing large volumes of data to construct predictive models for complex systems. In the field of materials science, machine learning has likewise demonstrated tremendous potential, including applications in molecular design and optimization [9], optimization of material processing techniques [10], material synthesis [11], defect detection [12], and property prediction [13]. In particular, for the property prediction of 2D semiconductor materials, researchers have employed a variety of machine learning algorithms, such as Gradient Boosting Decision Trees (GBDTs) [3], Support Vector Machines (SVMs) [14], and Multilayer Perceptrons (MLPs) [15], to train and evaluate models based on materials databases. By continuously optimizing these algorithms, it is possible to improve both the efficiency and accuracy of property prediction for 2D semiconductor materials. This facilitates fast and precise prediction of material properties, thereby accelerating the transition of 2D semiconductor materials from theoretical studies to practical applications, and advancing the development of next-generation high-performance 2D semiconductor devices, and ultimately providing a robust material foundation for current and future technological innovations.

This review aims to provide a comprehensive overview and summary of recent advances in the application of machine learning techniques for the prediction of properties in 2D semiconductor materials. First, the fundamental properties and application potential of 2D semiconductor materials are briefly introduced, followed by an illustration of traditional prediction approaches and emerging ML-based methods, through which the importance and challenges of property prediction in 2D semiconductors are indirectly elucidated. Subsequently, the review focuses on summarizing recent progress in the use of ML algorithms for predicting various properties of 2D semiconductor materials. This includes predictions of electronic structure properties such as bandgaps and magnetism, as well as other critical characteristics, including thermal conductivity, optical properties, mechanical performance, carrier mobility, and chemical stability. The article analyzes and compares the experimental methodologies and datasets employed by different research groups in recent years, demonstrating the predictive performance of various ML models for different material properties. In the concluding section, the review systematically summarizes the contributions of machine learning to the field of 2D semiconductor material property prediction, with particular emphasis on its advantages in improving efficiency and accuracy. Additionally, it discusses the current challenges facing ML-based approaches, including data quality and standardization, limited model interpretability, and insufficient cross-system generalization. To address these issues, several potential solutions are proposed, such as standardized materials databases, physics-informed machine learning, and the integration of machine learning with experimental and theoretical methods. Finally, the article offers perspectives on future trends in the application of machine learning for property prediction in 2D semiconductor materials.

In this review, we focus specifically on machine learning for 2D semiconductors, rather than broader 2D materials or general materials databases. This targeted scope allows us to systematically analyze the unique electronic and magnetic properties of 2D semiconductors and the corresponding ML prediction strategies, providing tailored guidance for researchers in the field. Unlike existing reviews organized solely by ML algorithms, we adopt a property-centered framework that structures the discussion around core prediction tasks, including bandgap, magnetic properties, and other key physical characteristics. For example, bandgap prediction is presented according to the evolution of ML models (from traditional methods to deep neural networks), while magnetic property prediction is discussed from the perspective of ML-driven advances in prediction efficiency, accuracy, materials discovery, and reproducibility. This structure clarifies research trends and key breakthroughs, while enabling readers to quickly locate relevant information and understand the strengths and limitations of different methods for specific tasks. Finally, we provide a critical overview of current challenges and future directions, including dataset expansion and standardization, physics-informed machine learning models, transfer learning-enabled multimodal fusion strategies, and the synergistic integration of machine learning with experimental techniques and traditional theoretical calculations, thereby offering a balanced and forward-looking perspective for advancing ML applications in 2D semiconductor research. The specific content arrangement of this review is shown in Table 1.

## 2. Fundamental Properties of 2D Semiconductor Materials and Prediction Methods

The key fundamental properties of 2D semiconductor materials span multiple dimensions, including the electronic structure, thermal conductivity, optical response, mechanical behavior, and chemical stability. Each property parameter not only reflects the underlying physical mechanisms at the microscopic scale but also directly determines the material’s application potential at the macroscopic device level. Typically, electronic properties such as bandgap and magnetism govern the core functionalities of 2D semiconductors in field-effect transistors, spintronic devices, and photodetectors. In contrast, properties such as thermal conductivity, mechanical flexibility, and optical absorption coefficients are critical for thermal management, flexible and wearable electronics, and photoelectric conversion efficiency. Therefore, the systematic prediction of these key physical properties has become one of the central approaches for advancing both theoretical understanding and practical engineering of 2D semiconductor materials. At present, property prediction methodologies for 2D semiconductor materials have evolved from traditional physics-based modeling approaches, including density functional theory (DFT), molecular dynamics (MD), and first-principles calculations, to data-driven intelligent strategies centered around machine learning. The latter includes a wide range of algorithmic frameworks, such as regression models, classification models, deep neural networks, and graph neural networks, which enable highly accurate property prediction while significantly improving modeling efficiency and generalization capability across complex material systems. With the continuous expansion of materials databases and the rapid progress in algorithmic performance, ML-based predictive techniques are emerging as essential tools for high-throughput screening and performance optimization of 2D semiconductor materials.

### 2.1. Properties of 2D Semiconductor Materials and Their Importance

Due to their unique physicochemical properties, including tunable bandgaps, intrinsic magnetism, excellent thermal conductivity, remarkable optical characteristics, outstanding mechanical performance, high carrier mobility, and good chemical stability, 2D semiconductor materials provide a robust foundation for applications across numerous domains and demonstrate great application potential for state-of-the-art science and technology.

Among the various properties, bandgap and magnetism are considered key performance indicators of 2D semiconductor materials, playing a vital role in their applications in the fields of electronics, optoelectronics, and spintronics. The bandgap governs the material’s optical absorption and emission characteristics, as well as its electronic behavior in semiconductor devices. Accurate prediction of bandgap values enables rapid screening of materials suited for specific optoelectronic devices such as solar cells, light-emitting diodes, and photodetectors, as well as semiconductor components including transistors and integrated circuits, thereby accelerating the development and deployment of novel materials and devices. Furthermore, the bandgap can be effectively tuned via several approaches, including strain engineering, external electric fields, atomic doping, and the construction of heterostructures [16], to meet research requirements. On the other hand, magnetism refers to the response of materials to a magnetic field, which primarily originates from the ordered alignment of intrinsic magnetic moments. Depending on the arrangement and interaction of these moments, materials may exhibit various magnetic behaviors, including ferromagnetism, antiferromagnetism, ferrimagnetism, and paramagnetism [17]. Predicting magnetic properties is crucial for designing multifunctional devices with magnetoelectric or spin-valley coupling, offering new avenues for quantum information processing and low-power electronics [18]. Beyond bandgap and magnetism, 2D semiconductor materials exhibit a range of other critical physical properties, including high thermal conductivity, excellent optical response, superior mechanical flexibility, and high carrier mobility. Systematic prediction of these characteristics enables researchers to evaluate the potential of candidate materials for applications in thermal management, optoelectronic conversion, flexible device fabrication, and energy modulation. For instance, thermal conductivity prediction facilitates the development of high-efficiency thermal management materials [19]; optical property prediction supports the structural design of optoelectronic devices [20]; and mobility prediction provides a theoretical foundation for realizing high-performance electronic devices [21].

In summary, theoretical predictions of the multidimensional physical properties of 2D semiconductor materials not only accelerate the discovery and screening of novel materials but also provide deep insights into the relationships between structure and properties. Furthermore, it also facilitates early-stage design of device functionalities and promotes the development of high-performance, low-power, and integrable devices. Property prediction methodologies have emerged as a critical bridge linking fundamental theory with practical engineering applications and are recognized as one of the key directions for the future advancement of materials science [22].

### 2.2. Conventional Methods for Predicting the Properties of 2D Semiconductor Materials

Due to their outstanding physicochemical properties and potential applications, 2D semiconductor materials have garnered considerable research attention in the field of materials science. As investigations deepen, the efficient and accurate prediction of their key properties has become a critical step in advancing the discovery, design, and application of novel materials. Traditional predictive approaches, such as density functional theory (DFT), molecular dynamics (MD) simulations, and Monte Carlo methods, have played a pivotal role in the property prediction of 2D semiconductor materials. For example, DFT calculations have been widely employed to investigate the electronic structure, adsorption behavior, and interfacial properties of various 2D layered materials, providing important theoretical guidance for material design and functional optimization [23,24]. These methods offer a high degree of physical interpretability, with outcomes that are closely correlated with the material’s microscopic structure and fundamental physical laws and enable the construction of multiscale correlation models linking atomic-scale features to macroscopic performance. These methods thus offer both generality and systematic insight, serving as powerful theoretical tools for uncovering the underlying mechanisms governing the formation of material properties and providing critical guidance for experimental exploration [25].

However, despite their advantages in predicting the properties of 2D semiconductor materials, the limitations of traditional methods have become increasingly prominent. These approaches are typically computationally intensive and time-consuming, particularly when dealing with material systems at a large scale or in high dimensions, making them less suitable for high-throughput screening with decreased computational efficiency. Furthermore, their reliance on prior knowledge and empirical parameters limits their predictive capability when applied to entirely new material systems or unknown structures. Specifically, for materials with significant structural differences and lacking historical data, traditional methods often struggle to adapt model architectures in real time, leading to delayed predictions or reduced accuracy. Additionally, traditional methods are primarily based on linear or quasi-linear approximations, which constrain their ability to capture strong nonlinear couplings or latent microstructural evolution principles embedded in complex materials systems [26].

Consequently, against the backdrop of the continuous emergence of new materials, there is an urgent need to develop new predictive strategies that are more efficient, flexible, and adaptable. Such approaches are indispensable for overcoming the limitations of traditional methods in terms of generalizability, efficiency, and ability to handle complex systems, thereby accelerating the discovery, optimization, and application of next-generation 2D semiconductor materials.

### 2.3. Machine Learning-Based Prediction of Properties for 2D Semiconductor Materials

Machine learning has experienced rapid development. ML represents a critical intersection between computer science and statistics, forming a core component of both AI and data science domains. Machine learning focuses on developing algorithms and models that enable computer systems to automatically learn from data and improve their performance through experience [27]. In the field of materials research, particularly in the prediction of properties of 2D semiconductor materials, ML has begun to play an increasingly important role. Specifically, machine learning is considered a promising strategy for addressing the inherent limitations of traditional computational approaches to 2D semiconductor material property prediction [28,29].

With the continuous advancement of research into the property prediction of 2D semiconductor materials, the machine learning algorithms adopted have evolved from single models to ensemble algorithms. Concurrently, the application of neural networks for property prediction has increasingly emerged. Initially, ML predominantly relied on single models such as linear regression [30], decision trees [31], and support vector machines (SVMs) [14]. These algorithms construct predictive models based on specific mathematical models or assumptions. Using different philosophies and methodologies, every single algorithm is trained on datasets to capture underlying patterns and make predictions with learned models. Due to their ease of implementation, computational efficiency, and interpretability, these single algorithms can offer satisfactory performance when the data adheres to the assumptions inherent to the algorithm. However, as research problems grow in complexity, particularly with the introduction of high-dimensional or nonlinear data, these single models encounter limitations such as overfitting, reduced efficiency, and increasing computational overhead. To enhance predictive performance, the ML community has increasingly turned toward ensemble algorithms. The fundamental principle of ensemble algorithms is to combine the predictions of multiple base learners to generate a final prediction, thereby mitigating the limitations of individual models and improving overall predictive performance for 2D semiconductor materials. One of the advantages of ensemble algorithms lies in their ability to reduce both bias and variance, thereby enhancing the generalization capacity, stability, and robustness of predictive models, particularly when dealing with noisy or complex datasets. Furthermore, ensemble algorithms exhibit strong fault tolerance by aggregating the strengths of multiple learners, which helps offset the weaknesses of any single model [32]. Nonetheless, ensemble algorithms also come with downsides in that the increased model complexity demands extensive data and computational resources to train multiple base learners. Furthermore, they depend on effective feature extraction and thus need hand-crafted features or feature selection strategies, and may underperform when faced with highly intricate nonlinear relationships. To address these issues, neural networks have emerged as a powerful alternative due to their exceptional capabilities in automated feature learning and modeling complex data structures, reducing the need for manual feature engineering and demonstrating strong performance in parsing multidimensional data. The evolution from single algorithms to ensemble algorithms, and subsequently to neural networks, reflects the growing maturity of ML applications in the field of 2D semiconductor materials property prediction. This technical progression has enabled the development of multidimensional and high-precision predictive modeling frameworks, offering systematic and intelligent solutions to increasingly complex challenges in materials research and engineering applications [33,34].

With the increasingly widespread application of machine learning in the property prediction of 2D semiconductor materials, researchers have increasingly focused on the advantages of this approach in terms of prediction efficiency, accuracy, and other performance metrics.

Machine learning enables a more efficient investigation into the property prediction of 2D semiconductor materials. Research in this field often involves large-scale high-dimensional datasets, encompassing atomic structures, electronic properties, mechanical performance, and more. Machine learning techniques, endowed with powerful data processing capabilities, incorporate a variety of highly efficient algorithmic models. For instance, there is Bayesian optimization, which is capable of identifying better solutions with fewer function evaluations [35], and deep learning models that capture underlying features through hierarchical abstract representations and thereby maintain high predictive performance even in complex multivariate environments [36]. These algorithms have substantially enhanced research efficiency.

Machine learning has demonstrated a remarkable capacity to enhance predictive accuracy. Owing to the limitations of computational resources and experimental techniques, and the introduction of defects and impurities during material synthesis, conventional experimental methods often fail to provide sufficiently precise data. By training on datasets of known materials, machine learning models can capture the intricate relationships between material properties and their structural characteristics, uncovering the intrinsic physical mechanisms and predicting the performance of novel 2D semiconductor materials with improved accuracy. Furthermore, as the volume of available data continues to grow, the predictive capability of such models can be continually enhanced. For instance, the team at the Advanced Energy Research Center of Kyoto University, Japan, employed a random forest algorithm to identify the most influential input variables for prediction from a large dataset, achieving high-precision predictions of valley polarization. This approach significantly improved predictive accuracy while reducing the time and cost of trial-and-error experimentation, thus allowing researchers to more precisely screen materials with potential application value [37,38].

Machine learning further facilitates the discovery and innovation of new materials. The reliance on trial-and-error experiments and theoretical calculations, coupled with the unique physicochemical properties of 2D semiconductor materials and their high sensitivity to fabrication processes, has limited innovation and the discovery of new 2D semiconductor materials. However, machine learning can assist researchers in discovering entirely new material systems, even materials that have not yet been predicted by theory, by analyzing vast datasets, conducting a global search of potential material combinations, guiding the optimization of synthesis routes, and integrating interdisciplinary knowledge, thereby effectively promoting material discovery. For example, the research team at Northwestern Polytechnical University concluded that machine learning can identify 2D semiconductor materials with targeted properties from a massive pool of candidate materials through efficient screening, performance prediction, and synthesis optimization [14]. In terms of material innovation, machine learning leverages efficient data analysis and pattern recognition to extract new material design principles from deep learning on existing material databases, even uncovering novel phenomena beyond the predictive scope of traditional theories [39]. Machine learning substantially reduces the cost and time of trial-and-error, while enhancing research precision and innovation capacity, thereby accelerating the transformation of novel materials from conceptualization to practical application.

Machine learning approaches have also enhanced the reproducibility and transparency of research. In traditional methodologies, low research standardization resulting from lab-specific conditions, manual operations, and limited data sharing often results in poor reproducibility; moreover, insufficient documentation and disclosure of experimental details and data analyses reduce research transparency. Differently, machine learning renders research results more verifiable and reproducible by establishing clear data-processing workflows and constructing standardized datasets. For example, the team at Fudan University presented a comprehensive account of the implementation process of machine learning for predicting the properties of 2D semiconductor materials, including the complete experimental dataset, detailed parameters, and procedural steps, thereby facilitating experimental reproduction by other researchers for further investigations [40]. Furthermore, the Technical University of Denmark proposed two representation methods, ENDOME and RAD-PDOS, which accurately extract electronic state fingerprints from DFT calculations for predicting the GW quasiparticle energy corrections of individual electronic states in 2D semiconductor materials. By employing a gradient boosting algorithm to construct a fingerprint–machine learning model, they developed a high-precision band structure model capable of accurately predicting GW electronic state energies for 2D semiconductor materials at low computational cost. They released in detail the machine learning methodologies and input features, the complete GW quasiparticle energy dataset of 2D semiconductor materials, and the corresponding Python code used in the prediction [41]. The standardization and easy disclosure of these data and methods increase the verifiability of research outcomes and foster scholarly communication and collaboration to further accelerate progress in the field of 2D semiconductor materials research.

In summary, with the iteration of machine learning prediction methods from single algorithms to ensemble approaches and the incorporation of neural networks, machine learning can effectively integrate the strengths of multiple models to further enhance predictive performance, with its advantages being more prominent when processing large-scale and complex datasets. From the perspective of predictive capability, the application of machine learning in forecasting the properties of 2D semiconductors not only markedly improves research efficiency and predictive accuracy but also provides strong support for the design and discovery of new materials, while enhancing the reproducibility and transparency of research. Machine learning offers a novel pathway for property prediction of 2D semiconductors and holds great potential to further advance the development of research in this field.

To provide a clearer understanding of the differences between conventional computational methods and machine learning approaches in 2D semiconductor property prediction, their representative methods, computational efficiency, physical interpretability, generalization capability, and typical applications are comparatively summarized in Table 2.

## 3. Research Progress in Predicting the Key Properties of 2D Semiconductor Materials

**Figure 1:**
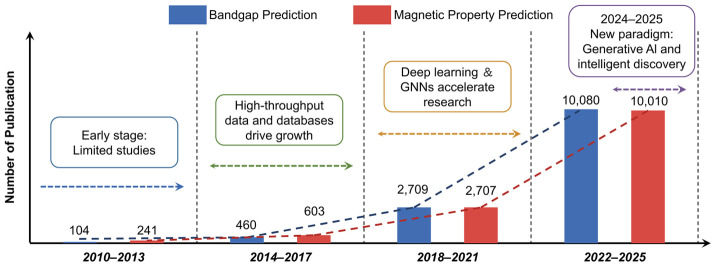
Evolution of machine learning research on bandgap and magnetic property prediction in 2D materials. Publication trends were estimated using keyword-based searches in Google Scholar with the keywords “2D materials” combined with “machine learning” and “bandgap” or “magnetic properties”.

Bandgap and magnetism are two highly significant properties of 2D materials. Research on the machine learning-driven prediction of bandgap and magnetism in 2D materials has witnessed a booming development trend over recent years (Figure 1). It can therefore be reasonably concluded that among the various physical characteristics of 2D semiconductor materials, bandgap and magnetism have garnered extensive attention by virtue of their fundamental importance and broad application prospects. Bandgap properties directly influence electronic and optoelectronic device performance, carrier transport, and optical absorption behaviors [42], while magnetic properties are closely related to spintronics and emerging quantum functional materials [43]. In addition, bandgap prediction has benefited from relatively mature databases and research frameworks, such as 2DMatPedia and C2DB [44,45,46]. Meanwhile, magnetic property prediction has recently become an important research direction because of its significance for high-throughput screening and novel magnetic material discovery [47]. Therefore, this review provides more detailed discussions on bandgap and magnetic property predictions, while other important physical properties, including thermal conductivity, optical properties, mechanical properties, carrier mobility, and chemical stability, are discussed more concisely in this section.

### 3.1. Bandgap Prediction

By predicting the bandgap values of different materials, researchers can screen for materials suitable for specific applications. Consequently, bandgap prediction for 2D semiconductor materials has now been widely applied across various fields. In the field of electronics, the bandgap is a determining factor in the electrical conductivity of materials. A larger bandgap corresponds to higher electrical resistivity, making such materials ideal for high-resistance electronic components, whereas a smaller bandgap implies higher conductivity, rendering such materials suitable for low-resistance devices [48]. Therefore, precise bandgap prediction provides a theoretical basis for the rational design and optimization of electronic devices. In photovoltaic applications, the bandgap energy governs the wavelength range of light absorption [49]. Reliable bandgap prediction enables the rapid and accurate selection of 2D semiconductors with optimal optical characteristics for solar cell fabrication. In other energy storage systems, the bandgap exerts a crucial influence on ion diffusion dynamics and electron transport behavior, which collectively determine the charge–discharge performance and cycling stability of batteries [50]. Hence, bandgap prediction serves as an effective preliminary screening tool for evaluating the electrochemical suitability of candidate materials. Beyond these applications, bandgap prediction also plays an essential role in photocatalytic water splitting for hydrogen evolution [51], guiding the rational design of high-efficiency photocatalysts. Nevertheless, as discussed earlier, conventional approaches for property prediction in 2D semiconductors, which rely on experimental trial-and-error or first-principles simulations, are often time-consuming, resource-intensive, and computationally demanding, thereby posing significant challenges to large-scale material screening.

This section integrates recent data-driven findings from various research groups employing machine learning to predict the bandgaps of 2D semiconductor materials. Machine learning comprises a class of algorithms that automatically analyze datasets to identify patterns and leverage these patterns to make predictions on unknown data. In bandgap prediction, machine learning plays a pivotal role. Its primary operational principle involves selecting appropriate machine learning models and using feature datasets derived from materials to be predicted for model training and testing. The trained models can then rapidly and accurately predict the bandgaps of similar materials. Researchers have explored both single machine learning algorithms and ensemble algorithms for bandgap prediction in 2D semiconductor materials. Moreover, the rapid advancement of neural networks has provided novel pathways for enhancing bandgap predictive accuracy in these materials.

#### 3.1.1. Single Algorithms for Bandgap Prediction

In the prediction of bandgaps for 2D semiconductor materials, certain studies have primarily relied on single algorithm frameworks, such as linear models and kernel-based approaches, utilizing simple elemental attributes (e.g., electronegativity and ionic radius) or structural descriptors (e.g., lattice constants) as predictive variables.

The representative linear models mainly include linear regression and logistic regression. These models predict the bandgap through a linear combination of descriptors. Typically, physicochemical features such as atomic electronegativity and atomic radius are employed to construct feature matrices, while regularization techniques are applied to identify key variables. Subsequently, model parameters are optimized using algorithms such as the least squares method or gradient descent. Linear models exhibit high computational efficiency and strong interpretability, making them particularly suitable for small datasets. However, constrained by the linearity assumption, their capability to capture nonlinear correlations remains limited. Linear regression fits an optimal line or hyperplane to describe the linear dependency between independent and dependent variables, thereby minimizing prediction error. For example, in 2023, in a comparative study conducted by Cheng et al. on machine learning models for predicting 2D semiconductor bandgaps [52], the linear regression model achieved a root mean square error (RMSE) close to zero on the training set, indicating excellent fitting performance. Nevertheless, its predictive accuracy on the test set declined substantially, suggesting overfitting to the training data. Moreover, a considerable number of outliers were observed in the prediction results, implying that the model’s stability and generalization performance are highly dependent on dataset partitioning. Logistic regression, in contrast, introduces a sigmoid function to transform the linear combination of input features into probabilistic outputs, thereby modeling the likelihood of specific categorical outcomes. As a generalized linear model primarily used for binary classification, it is computationally efficient and suitable for cases where the relationship between descriptors and target variables is approximately linear. For instance, in 2018, a joint research team from the University of Sydney and the University of California, Santa Barbara developed a logistic regression-based binary classifier to distinguish between materials with direct and indirect bandgaps [53]. This approach not only provided classification outputs but also yielded probabilistic confidence estimates for each class. By minimizing classification errors through parameter optimization, the model achieved an accuracy of 89%, and the results showed strong consistency with first-principles hybrid functional (HSE) calculations. In summary, both linear regression and logistic regression, as representative linear modeling paradigms, offer distinct advantages in interpretability, computational efficiency, and model transparency. Despite their inherent limitations in capturing nonlinear dependencies, these models have demonstrated practical utility in bandgap prediction for 2D semiconductor materials, particularly in scenarios involving limited data availability and high demands for explainability.

Another important class of single machine learning algorithms applied to bandgap prediction in 2D semiconductors includes support vector machines (SVM), kernel ridge regression (KRR), and Gaussian process regression (GPR). In this review, these algorithms are collectively categorized as kernel-based machine learning models. By employing kernel functions, these models project low-dimensional features into high-dimensional spaces, thereby effectively capturing the nonlinear relationships between the bandgap and material descriptors. Typical kernel functions, such as the radial basis function (RBF) and polynomial kernel (PK), are frequently used to handle the complex correlations between atomic structural parameters (e.g., bond length and interlayer distance) and the electronic bandgap. These models generally perform well in small-sample scenarios but exhibit sensitivity to the choice of kernel function and hyperparameter tuning. In 2020, Zhu et al. employed three regression algorithms, namely linear regression (LR), random forest regression (RFR), and support vector regression (SVR), to predict the electronic properties of 2D semiconductor materials using data obtained from two density functional theory (DFT) methods, Perdew–Burke–Ernzerhof (PBE) and Heyd–Scuseria–Ernzerhof (HSE). Based on PBE results and elemental characteristics, three distinct feature sets were constructed for model training, with the aim of predicting the valence band maximum (VBM), the conduction band minimum (CBM), and, ultimately, the bandgap defined as the difference between VBM and CBM. The results showed that, when feature set III was adopted, the SVR model exhibited superior performance, achieving RMSE values of 0.13 eV for VBM, 0.08 eV for CBM, and 0.09 eV for the bandgap, notably outperforming LR (0.15 eV, 0.09 eV, 0.09 eV respectively) and RFR (0.25 eV, 0.18 eV, 0.18 eV respectively). Moreover, SVR achieved the lowest mean absolute percentage error (MAPE) of 4.93%, compared with 5.5% for LR and 7.44% for RFR. These findings demonstrate that machine learning models can accurately predict both band-edge positions and bandgap values, and thus provide an efficient route for screening 2D semiconductors with target bandgaps, and can be extended to more complex systems such as alloys, thereby offering valuable insights for the design of materials in optoelectronic and photocatalytic applications [54]. Previous studies [52] have further shown that SVM-based models yield highly reliable predictions for semiconductors with bandgaps in the 0–3 eV range, while their accuracy slightly declines for wide-bandgap materials that are within the 4–5 eV range. Similarly, kernel ridge regression (KRR), another kernel-based method, has been applied to the bandgap prediction of 2D semiconductors. In 2018, Singh et al. utilized KRR to estimate the bandgaps of MXene materials, a family of 2D transition metal carbides and nitrides, by training on DFT-calculated datasets incorporating 158 compositional and structural descriptors. The model achieved an RMSE of 0.19 eV and a determination coefficient (R^2^) of 0.68 on the primary feature test set, indicating that KRR can accurately capture the electronic properties of 2D semiconductors [55]. Although 2D perovskite materials share the typical semiconducting features of two-dimensional systems, their structural frameworks differ markedly from those of double perovskites. Nevertheless, both material families pursue the same objective, namely the construction of mapping relationships between structure and property for precise bandgap prediction, and both can benefit from the efficiency and accuracy of machine learning methods. Hence, advances in bandgap prediction for double perovskites can offer a valuable reference for research on 2D semiconductors. For instance, in 2015, Pilania et al. investigated bandgap prediction in double perovskite materials using linear least-squares fitting (LLSF) and kernel ridge regression (KRR), with model performance evaluated via cross-validation. When employing a four-dimensional descriptor as the optimal feature set, the LLSF model yielded a RMSE of approximately 1.1 eV with an R^2^ of 0.5, whereas the KRR model exhibited significantly better performance, achieving RMS of 0.306 eV and R^2^ of 0.963 on the training set, and RMS of 0.501 eV, R^2^ of 0.897 on the test set, thereby demonstrating both high fitting accuracy and strong generalization capability [56]. Beyond SVM and KRR, GPR represents another critical kernel-based approach for bandgap prediction. A major advantage of GPR lies in its probabilistic nature, which provides confidence intervals for each predicted value. As reported by Singh and co-workers [55], the GPR model achieved an RMSE of 0.06 eV and R^2^ of 0.97 on the main training set, and outperformed other models (KRR, SVR, and Bagging) on the test set with the lowest RMSE of 0.14 eV and the highest R^2^ of 0.83. These results underscore the efficiency and accuracy of GPR in predicting the bandgaps of 2D semiconductor materials.

**Figure 2:**
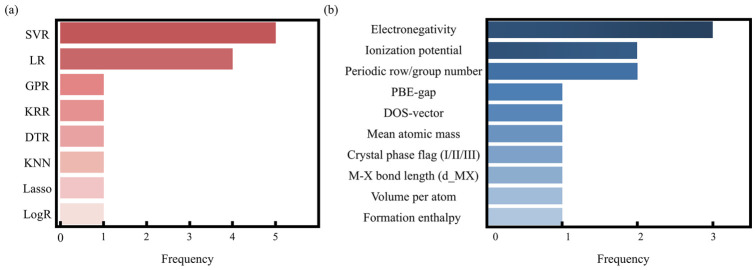
Ranking of single machine learning models and corresponding features used for predicting the bandgap of 2D semiconductors. (**a**) Model usage frequency; (**b**) feature usage frequency.

Based on representative studies [52,53,54,55,56], we conducted a quantitative statistical analysis of the usage frequency of individual machine learning models and the usage frequency of different features in bandgap prediction, as summarized in Figure 2. Figure 2a shows the distribution of model usage, and Figure 2b illustrates the distribution of feature usage. The statistics show that SVR is the most frequently employed model, followed by LR. This trend can be attributed to several factors. SVR is widely adopted because it exhibits strong robustness in small-sample settings, involves a relatively small number of hyperparameters, and effectively captures the nonlinear characteristics of residuals with its RBF kernel. LR is also extensively used owing to its essentially parameter-free form, high interpretability, and strict linear modeling capacity, which together lead to efficient training, concise interpretation, and clear guidance for subsequent model refinement. By contrast, other methods are less frequently used due to limitations such as restricted dimensional scalability (such as KNN), high computational cost (such as KRR), or mismatch with the regression nature of the task (such as LogR). With respect to feature selection, electronegativity, ionization potential, and position in the periodic table are most frequently employed, since they are easily accessible and strongly correlated with bandgap values. In contrast, high-cost descriptors such as the PBE gap and DOS vector, as well as features with limited applicability such as the phase_flag, are used far less frequently.

Single algorithms, including linear models and kernel-based methods, typically feature simple architectures, low computational and memory costs, and strong physical interpretability, allowing for a transparent understanding of the mechanism underlying their predictions. These models can even achieve near-optimal performance when applied to small datasets, relatively simple prediction tasks, or problems requiring high model interpretability. However, when confronted with high-dimensional or intrinsically complex datasets, their performance often deteriorates, exhibiting limited generalization capability and a clear tendency toward overfitting.

#### 3.1.2. Integrated Model for Bandgap Prediction

Ensemble learning is a machine learning strategy that enhances predictive accuracy and generalization capability by combining multiple base learners and integrating their respective strengths. It effectively mitigates the limitations of single algorithms, such as high variance and overfitting. Ensemble learning methods are typically categorized into Bagging, Boosting, and Stacking. Among them, Bagging and Boosting have been most widely applied in the field of 2D semiconductor materials property predictions, and substantial research progress has been achieved. This section focuses on the specific applications of these two methods in bandgap prediction of 2D semiconductor materials.

The Bagging method constructs multiple sub-datasets by randomly sampling the training set and trains an independent base learner on each subset. The final prediction is obtained by averaging or voting over the outputs of all base learners. This approach enables parallel computation across learners, effectively reducing the variance of the final model while maintaining robustness. However, its computational cost increases significantly due to the need to train multiple models [57]. Overall, Bagging remains a highly valuable approach in the field of 2D materials property prediction. Among these, representative Bagging tree models, including random forests (RF), Extra Trees, and Bagging regression, construct ensembles of decision trees through recursive feature splitting and are particularly adept at handling high-dimensional and heterogeneous data. When applied to datasets containing multi-source descriptors such as material composition (for example, elemental composition ratios), structural symmetry, and strain parameters, tree-based models can automatically capture complex feature interactions, thereby accelerating progress in bandgap prediction for 2D semiconductors. For instance, in 2022, Lifeng Dong et al. developed four random forest regression models to predict the bandgaps of double perovskite oxides (DPOs), based on two types of feature sets (merged and initial) and two feature selection methods (Recursive Feature Elimination, RFE; and Univariate Feature Selection, UFS). The models were denoted as M1 (merged set, RFE, 3 features), M2 (initial set, RFE, 6 features), M3 (merged set, UFS, 20 features), and M4 (initial set, UFS, 20 features). Their performance on the test set showed R^2^ values of 0.932, 0.934, 0.910, and 0.947, and RMSE values of 0.196 eV, 0.193 eV, 0.225 eV, and 0.172 eV, respectively. Notably, Model M1 achieved a favorable balance between predictive accuracy and computational efficiency with fewer input features, making it particularly suitable for large-scale material screening. Furthermore, the RMSE values on both the training and test sets were below 0.2 eV, indicating that the RF model possessed excellent generalization capability and predictive accuracy [58]. This study demonstrated that random forest models not only achieve high accuracy and low error in bandgap prediction of 2D semiconductors but also offer flexible feature selection and strong generalization performance, highlighting their potential in large-scale materials discovery. In addition, similar performance has been observed in 2D nanomagnetic materials that share structural characteristics with 2D semiconductors. In 2024, Soumya Jyoti Ray et al. employed various machine learning algorithms, including linear regression, LASSO, decision tree, support vector machine, and random forest, to predict the bandgaps and other properties of 2D magnetic materials. Among all models tested, the random forest exhibited the lowest RMSE (0.22 eV) in predicting hybrid-functional (HSE)-based bandgap values derived from first-principles calculations, outperforming all other algorithms. This finding further underscores that the Bagging paradigm within ensemble learning provides a powerful and innovative framework for bandgap prediction in 2D semiconductor materials [59]. Beyond random forests, other tree models based on the Bagging strategy also exhibit strong performance. For example, Extra Trees achieves mean squared errors of 0.0005 and 0.0009 and a Pearson correlation coefficient above 0.998 on the test set in predicting the conduction and valence band structures of two-dimensional transition metal dichalcogenide alloys, indicating that tree-based models are capable of capturing band edge characteristics and thereby facilitating the prediction of band gaps and related electronic properties [60]. In addition, Li et al. compared multiple machine learning models for band gap prediction in ABO_3_-type perovskites, among which Bagging regression attains an R^2^ of 0.8569 on the test set, further supporting the effectiveness of the Bagging ensemble strategy in predicting electronic structures of complex material systems and providing a useful reference for its application to band gap prediction in two-dimensional semiconductors [61].

Boosting represents another major branch of ensemble learning. It constructs a high-performance model with low overfitting and strong adaptability by iteratively training base learners and combining them through weighted aggregation and adaptive reweighting. However, boosting methods are highly sensitive to noise and outliers and generally incur higher computational costs; their overall performance is also mainly dependent on the choice of base learners [62]. Among boosting algorithms, the Gradient Boosted Decision Tree (GBDT) stands out as one of the most representative and effective methods, demonstrating excellent predictive capability in the bandgap prediction of 2D semiconductor materials. For instance, in 2021, a collaborative research team from Shenyang Ligong University and Changchun Normal University published a study employing four machine learning algorithms (GBDT, RF, SVR, and MLP) to predict the bandgaps of 2D semiconductors. The model performance was evaluated using the coefficient of determination R^2^ and RMSE. Results showed that GBDT achieved superior regression accuracy, with an R^2^ exceeding 0.90 and an RMSE of 0.24 eV, outperforming RF (RMSE of 0.27 eV), SVR (RMSE of 0.41 eV), and MLP (RMSE of 0.43 eV). These findings highlight the outstanding predictive performance of Boosting-based ensemble algorithms, particularly GBDT, in bandgap estimation for 2D semiconductors [31]. Furthermore, in another study [59], XGBoost, an optimized implementation of gradient boosting, exhibited a high R^2^ of 0.91 and a low RMSE of 0.23 eV in predicting bandgap values obtained from hybrid functional (HSE) first-principles calculations. Such evidence further substantiates that Boosting-based ensemble learning not only surpasses single-model approaches in predictive accuracy but also holds significant potential for bandgap prediction in 2D semiconductors and structurally analogous materials.

**Figure 3:**
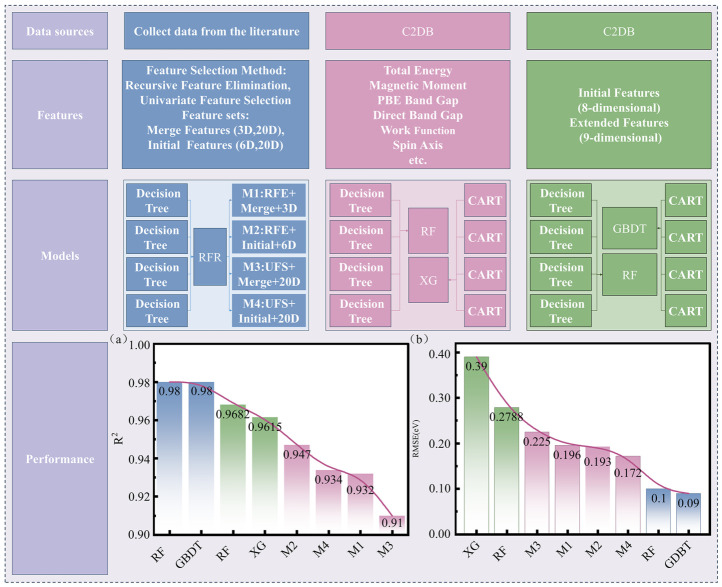
Summary of four aspects of ensemble algorithms for bandgap prediction in 2D semiconductors, encompassing data sources, feature representations, model architectures, and performance comparison. (**a**) Comparison of R^2^ among different ensemble algorithms for bandgap prediction; (**b**) comparison of RMSE among different ensemble algorithms for bandgap prediction. Note: In the bar charts of Figure 3a,b, the blue-colored data is from Ref. [31], the pink data from Ref. [58], and the green data from Ref. [59].

Drawing on representative studies [31,58,59], we outline the ensemble learning framework for bandgap prediction from four perspectives, as shown in Figure 3. C2DB constitutes the primary data source. For feature engineering, researchers typically construct feature sets of different dimensionalities and compare their performance to identify an appropriate representation. Model development is commonly based on tree-structured learners, which serve as the backbone of most ensemble algorithms. Performance statistics, summarized in Figure 3a, show that RF [31] and GBDT achieve the highest R^2^ values, whereas M3 performs the worst. The comparison of RMSE values in Figure 3b further confirms that RF [31] and GBDT exhibit the best predictive accuracy, while XG and RF [59] yield the poorest results. Overall, the combined evidence in Figure 3a,b indicates that RF [31] and GBDT provide the most reliable bandgap predictions, whereas M3 demonstrates the lowest performance.

This performance discrepancy can be rationalized from three perspectives: algorithm design, feature representation, and dataset characteristics. RF and GBDT benefit from ensembles of multiple decision trees, which endow them with strong nonlinear mapping capability, flexible handling of complex data distributions, and high fitting accuracy. The inclusion of bandgap values calculated without spin–orbit coupling (SOC) in the feature space [31] further enhances model performance, as reflected by increased R^2^ and reduced RMSE. Although M3 is also built on RF, its use of UFS for feature selection together with a merged 20-dimensional feature set introduces substantial feature redundancy and a tendency toward overfitting, and its failure to effectively exploit doping-related descriptors [58] leads to the poorest overall performance. In addition, the model proposed by Kar et al. [59] achieves a higher R2 compared with that of Liu et al. [58]; however, it yields a larger RMSE. We hypothesize that this arises because the former relies on DFT-HSE data and performs global modeling over a wide energy window of 0 to 6 eV for structurally diverse 2D magnetic materials, where larger label variance, higher noise levels, and stronger heterogeneity make accurate prediction more difficult. By contrast, the latter conducts local regression within a narrower bandgap range of approximately 2 to 3 eV for double perovskite oxides with relatively uniform structures, which facilitates achieving lower prediction errors.

Although ensemble algorithms generally outperform single models in handling large and complex datasets, they suffer from increased model complexity, limited interpretability, and the need for extensive hyperparameter tuning. In contrast, neural network models possess strong representational capacity, enabling automatic feature learning and the capture of complex nonlinear relationships. These characteristics make neural networks particularly suitable for tackling intricate prediction tasks and provide promising avenues to overcome the current challenges in bandgap prediction of 2D semiconductor materials.

#### 3.1.3. Neural Networks Used for Bandgap Prediction

Neural networks consist of numerous simple processing units interconnected through weighted links, enabling parallel processing and efficient computation. Their nonlinear activation functions provide these models with the capability to capture complex nonlinear relationships [63]. In the bandgap prediction for 2D semiconductor materials, neural networks can be employed as standalone predictors or integrated into ensemble learning frameworks to further enhance predictive accuracy and computational efficiency.

Common neural network architectures employed in this field include deep convolutional neural networks (CNNs), Bayesian neural networks (BNNs), and crystal graph convolutional neural networks (CGCNNs), each offering distinct advantages depending on the prediction task. For instance, CNNs excel at capturing lattice structural features and interatomic interactions, BNNs can provide uncertainty estimates alongside predictions, and CGCNNs directly learn atomic coordination and topological features from crystal graph representations. For instance, Jianlin Cheng et al. developed a CNN-based high-throughput framework for bandgap prediction. This framework was trained on tens of thousands of data points generated by batch DFT calculations with diverse doping levels and configurations, and such high-quality and large-scale training data effectively improved the robustness of the model. In a 4 × 4 supercell system, the CNN achieved R^2^ of 0.9547 and RMSE of 0.09 eV, markedly outperforming traditional support vector machine (SVM, R^2^ of 0.0029, RMSE of 0.33 eV). For larger 5 × 5 supercell systems, CNN-based extended models maintained strong predictive performance: the VGG16 convolutional network (VCN) achieved R^2^ of 0.9212 and RMSE of 0.09 eV; the residual convolutional network (RCN) achieved R^2^ of 0.9124 and RMSE of 0.09 eV; and the cascaded convolutional network (CCN) achieved R^2^ of 0.9285 and RMSE of 0.16 eV. All these CNN-based models significantly outperformed the SVM with an RMSE of 0.51 eV [64], demonstrating the superior accuracy and scalability of neural networks for bandgap prediction in 2D semiconductor materials.

To achieve higher accuracy and stronger generalization in bandgap prediction for 2D semiconductors, researchers have increasingly explored hybrid ensemble strategies that integrate neural networks with other machine learning methods. Such hybrid models not only enhance learning efficiency under limited data conditions but also significantly improve the adaptability of models in complex material systems, thereby expanding their applicability across large-scale material spaces. For example, in 2021, Fronzi et al. employed a Bayesian neural network (BNN) in combination with active learning (AL) to predict the bandgaps of 2D semiconductors. The study first trained the BNN model using HSE06-calculated data and then iteratively introduced structures with high predictive uncertainty via AL, ultimately constructing a training set comprising 473 structures. This approach enabled the creation of a bandgap database covering approximately 2.2 million novel 2D van der Waals heterostructures (vdWHs) while relying on a limited number of DFT calculations. After five iterative rounds, the BNN model achieved R^2^ of 0.8, MAPE of 0.2%, RMSE of 0.44 eV, and MAE of 0.30 eV on the training set, providing both high predictive accuracy and a methodological basis for large-scale material design and high-throughput screening [65]. In 2023, a team from the University of Electronic Science and Technology of China proposed a hybrid modeling framework that integrated structural symmetry-based screening, DFT + U calculations, and neural networks. Regarding model performance, a shallow neural network based on gradient boosting regression (GBR) achieved an R^2^ of 0.917 and an MSE of 0.003 on the test set, substantially outperforming traditional models such as SVM (R^2^ of 0.566) and RF (R^2^ of 0.809). Furthermore, incorporating a few-shot learning-enhanced deep neural network significantly improved extrapolation capability for low-doping configurations (e.g., 1/128 and 2/128 doping concentrations), reducing prediction errors to within ±0.1 eV, compared with shallow models where errors could reach ±0.3 eV. This demonstrates the accuracy and efficiency of neural networks in bandgap prediction for 2D semiconductor materials, particularly highlighting their remarkable scalability and practical applicability under low-doping conditions and limited computational resources [66]. In 2024, Wey Yang Teoh et al. developed a bandgap prediction model for 2D semiconductors by combining crystal graph convolutional neural networks (CGCNN) with transfer learning (TL), based on the C2DB database. This framework enabled high-precision prediction of valence band maxima (VBM) and conduction band minima (CBM), allowing comprehensive bandgap estimation, while also elucidating the effects of different surface orientations and subtle atomic displacements on the band structure. Notably, the study extended the data from 2D to 3D materials, achieving R^2^ of 0.96 and RMSE of 0.20 eV on the 2D test set, and R^2^ of 0.89, RMSE of 0.27 eV on the 3D test set. This extension overcomes dimensional limitations in material property studies, although some discrepancies remain in 3D predictions, indicating the need for further optimization. This work represents the first comprehensive prediction of VBM and CBM energies across both 2D and 3D levels, providing deeper insight into material physicochemical properties and offering a new strategy for efficient and precise design of 2D semiconductors [67]. In 2025, Qionghua Zhou et al. constructed a machine learning model combining CGCNN and one-dimensional convolutional neural networks (1D CNNs) to predict bandgaps for both 2D semiconductors and 2D heterostructures. The model utilized matrix descriptors to capture interlayer interactions and incorporated transfer learning to enhance generalization. Compared with linear regression models based on PBE and HSE functionals, the hybrid network significantly improved predictive accuracy. The model achieved R^2^ > 0.97 and MAE < 0.16 eV on both training and test sets and maintained high accuracy on the validation set (R^2^ > 0.93, MAE < 0.16 eV). Using this framework, over 800 potential type-II vdWHs were identified from 99,681 possible heterostructures, establishing a new neural network modeling paradigm for performance prediction in 2D semiconductors [68]. From 2021 to 2025, with the continuous development of hybrid machine learning models, the prediction performance of 2D semiconductor bandgap has been steadily improved, which is reflected in an increase in R^2^ from 0.8 to more than 0.97 and a decrease in the prediction error from 0.44 eV to less than 0.16 eV. These advances show the great potential of hybrid neural network ensemble strategies in achieving high-precision, scalable, and transferable bandgap prediction.

**Figure 4:**
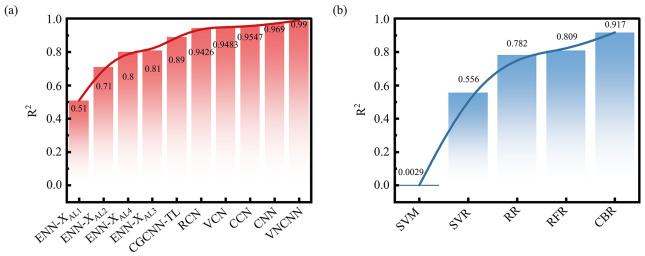
Performance (R^2^) comparison between (**a**) neural-network and (**b**) non-neural-network models for bandgap prediction in 2D semiconductors. (The data were sourced from references [65,66,67,68].).

Based on representative studies [65,66,67,68], we summarized the comparison of R^2^ values reported for neural network and non-neural network algorithms, as illustrated in Figure 4. Figure 4a shows the R^2^ performance of different neural network models, and Figure 4b presents the corresponding performance for non-neural network methods. According to Figure 4a, the R^2^ values of neural network algorithms span from approximately 0.51 to 0.99, with most values concentrated above 0.80, indicating a higher performance upper bound and relatively stable behavior. Figure 4b indicates that although some non-neural network methods achieve high R^2^ values, several algorithms yield notably poor performance. For example, SVM attains an R^2^ of only 0.0029 because its use of two-dimensional matrix-like inputs limits its ability to retain structural features, which impedes effective utilization of material-structure information. Taken together, within the scope of these studies, neural network models overall exhibit better performance than non-neural network methods. This advantage mainly derives from the capability of deep networks to capture complex nonlinear structural features through hierarchical representation learning and to maintain strong fitting ability in high-dimensional spaces. In addition, transfer learning enables favorable generalization in small-sample scenarios. Among these models, VNCNN demonstrates the best performance due to its deep convolutional architecture, the use of ReLU activations and the Adam optimizer, and the incorporation of effective regularization strategies.

**Figure 5:**
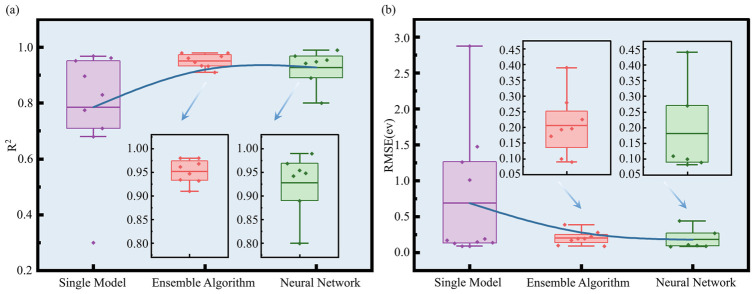
Performance comparison of single models, ensemble algorithms, and neural networks: (**a**) R^2^; (**b**) RMSE. (The data were sourced from references [31,52,53,54,55,56,58,59,60,61,64,65,66,67,68].) Note: The solid blue line shows the overall trend of model performance, with the arrow marking the zoomed-in inset panel.

To further visualize the overall differences among single models, ensemble algorithms, and neural networks in bandgap prediction, we surveyed the literature on machine learning-based bandgap prediction and summarized the reported R^2^ and RMSE values for these three categories, as shown in Figure 5. For the R^2^ metric in Figure 5a, the mean values of the three categories exhibit an overall increasing trend, and the slight decrease for neural networks arises from the relatively low performance of a few individual models with an R^2^ of 0.80. For the RMSE metric in Figure 5b, the mean values show a decreasing trend, which likewise reflects increasing predictive accuracy. These results indicate that, within current studies, single algorithms generally yield lower predictive accuracy. By contrast, ensemble learning, through the combination of multiple base learners and related strategies, achieves marked improvements in both R^2^ and RMSE, and its overall performance can, to some extent, already be considered comparable to that of neural networks.

In summary, this paper systematically summarizes the predictive performance of various machine learning models for predicting the bandgaps of 2D semiconductor materials, following the sequence of single algorithm models, ensemble algorithm models, and neural network-based approaches. The analysis highlights the remarkable advantages of neural networks in complex feature extraction and high-precision prediction, as illustrated in Figure 5. Future research may further investigate the integration of deep learning with other advanced techniques to expand the application scope of performance prediction for 2D semiconductor materials.

### 3.2. Magnetic Property Prediction

The magnetic properties of 2D semiconductor materials make them highly promising for applications in fields such as information storage, quantum computing, and spintronics. For example, by leveraging the ferromagnetism of 2D semiconductor materials, a high-density, low-power, non-volatile memory can be fabricated [69]. And by leveraging the antiferromagnetic properties of 2D semiconductor materials, it is possible to develop terahertz spin field-effect transistors with no stray fields and low power consumption to facilitate the development of communication chips [70]. Magnetic property prediction of 2D semiconductor materials refers to the use of theoretical calculations or experimental methods to predict whether 2D semiconductor materials possess magnetic properties and their specific magnetic types [71]. In exploring the application of machine learning in the field of magnetic property prediction for 2D semiconductor materials, researchers have focused on contributing to improvements in efficiency and accuracy, promoting the discovery and innovation of new materials, and enhancing the reproducibility and transparency of research. The following sections summarize the contributions made by various teams in this field over the past few years, organized around these key aspects.

#### 3.2.1. Research on Improving the Efficiency of Magnetic Property Prediction

Machine learning can quickly process and analyze large amounts of complex 2D semiconductor material data, automatically identifying key features and patterns that influence magnetism. Compared with traditional methods, this has significantly improved the efficiency of predicting the magnetism of 2D semiconductor materials. More efficient prediction of the magnetism of 2D semiconductor materials can bring greater convenience to research, and thus some researchers are dedicated to enhancing the efficiency of magnetic property prediction. This section summarizes the contributions made by different teams in improving the efficiency of magnetic property prediction for 2D semiconductor materials by combining various machine learning methods.

Prediction of key magnetic physical quantities is the mainstream approach for predicting the magnetic properties of 2D semiconductor materials. Key magnetic physical quantities include Hubbard parameters, magnetic ground states, and magnetic moments. Hubbard parameters represent the strength of Coulomb repulsive interactions between electrons within an atom and can help more accurately describe electronic behavior and material magnetism [72]. The magnetic ground state represents the magnetic configuration of magnetic materials at their lowest energy state, serving as the foundation for understanding the magnetic behavior of materials [73]. And the magnetic moment characterizes the orientation and response capability of magnetic materials or particles in a magnetic field [74].

##### Magnetic Property Prediction in 2D Semiconductor Materials

Researchers are gradually achieving rapid prediction of key magnetic parameters through the combination of machine learning and first-principles calculations. For example, in M. Modarresi’s team combined density functional theory (DFT) and machine learning techniques to predict the magnetic ground state and other properties of materials such as transition metal dichalcogenides. The team constructed a high-quality dataset based on DFT calculations and subsequently employed neural networks (NN), random forests (RF), and support vector regression (SVR) for comparative analysis. Among these models, neural networks exhibited the best predictive accuracy and reliability, enabling efficient prediction of Hubbard U parameters, magnetic exchange parameters, and magnetic phases. This approach significantly reduced the dependence on large-scale DFT calculations and improved the efficiency of magnetic property prediction for 2D semiconductor materials [75]. This work demonstrates the strong potential of combining machine learning with first-principles calculations for accelerating magnetic property prediction in two-dimensional materials. However, the prediction performance of such models still strongly depends on the quality and scale of DFT-generated datasets.

Subsequent research further focused on descriptor optimization and model generalization for magnetic property prediction. Jinlan Wang’s team developed an adaptive framework combining machine learning with high-throughput density functional theory (HT-DFT) to study the magnetic ground state prediction of 2D semiconductor materials, including ferromagnetic, antiferromagnetic, and non-ferromagnetic states. To improve prediction efficiency, the team designed crystal graph multilayer descriptors (CGMD) and adopted dimensionality reduction strategies to decrease feature complexity and accelerate model training. In terms of model structure optimization, the team selected a gradient-boosting classifier algorithm suitable for small-scale datasets and introduced an iterative feedback loop to continuously refine the model and enhance its generalization capability and prediction efficiency. After implementing these improvements, the method effectively extracts latent physical knowledge from the data and constructs mappings with corresponding properties to achieve excellent prediction performance [39]. This work highlights the importance of descriptor engineering and iterative optimization strategies in improving prediction efficiency and model performance for small-scale magnetic datasets. Nevertheless, the framework still relies heavily on high-throughput DFT-generated data.

Meanwhile, other studies further explored the integration of machine learning with high-throughput screening strategies for large-scale magnetic material discovery. Chao Xin’s team combined machine learning and high-throughput first-principles calculations (DFT) to study magnetic property predictions for 2D ferromagnetic materials (including 2D ferromagnetic semiconductor materials). The team first used multiple algorithms, such as K-nearest neighbors (KNNs) and support vector machines (SVMs), to classify magnetic and non-magnetic, ferromagnetic and antiferromagnetic materials. Subsequently, they employed random forest regression (RFR), gradient-boosted decision trees (GBDTs), extreme gradient boosting (XGBoost), and artificial neural network (ANN) to predict the net magnetic moment of 2D ferromagnetic semiconductors and other materials. To enhance the model’s generalization capability and computational efficiency, the method employed 10-fold recursive feature elimination combined with cross-validation (RFECV) to optimize the hyperparameters of all models, selecting 24 important features from 273 initial features, effectively reducing overfitting and computational complexity. On the other hand, DFT calculations took approximately 57,600 core hours, while training and screening machine learning classification and regression models took approximately 560 core hours, achieving a significant reduction in computational time and further improving the efficiency of magnetic property prediction for 2D semiconductor materials [76]. This work demonstrates that combining machine learning with high-throughput first-principles calculations can significantly improve prediction efficiency for magnetic properties of two-dimensional materials.

In summary, the above studies consistently highlight the critical role of machine learning in improving the efficiency of magnetic property prediction for 2D semiconductor materials. M. Modarresi’s team reduced the heavy reliance on high-cost DFT calculations by employing machine learning models, thereby cutting down both computational time and resources. Jinlan Wang’s team further accelerated the prediction process through customized feature descriptors and dimensionality reduction strategies, significantly improving model training and inference speed. And Chao Xin’s team achieved a more remarkable enhancement in efficiency by optimizing feature selection and model hyperparameters, which drastically reduced the overall computational cost. These works demonstrate that reasonable algorithm selection, targeted feature engineering, and systematic model optimization are effective ways to significantly boost the efficiency of magnetic property prediction. However, current studies still mainly rely on DFT-generated datasets. Although prediction efficiency has been significantly improved, many studies primarily focus on enhancing predictive accuracy and computational efficiency, while discussions regarding the physical significance and stability of the models remain relatively limited. Therefore, future research should further emphasize improvements in model robustness and physical interpretability while maintaining high prediction efficiency.

##### Magnetic Property Prediction in 2D Magnetic Materials

The magnetic property prediction of 2D magnetic materials is closely related to that of 2D semiconductor materials. The former aims to discover and design 2D materials with intrinsic magnetic properties, while the latter focuses on inducing or regulating magnetic properties in intrinsically non-magnetic 2D semiconductors through means such as stress and doping. These two categories of research share a high degree of methodological consistency, both relying on band structure manipulation, electronic structure analysis, and high-throughput screening techniques, and both benefiting from the efficiency and interpretability brought by the introduction of machine learning technologies. Therefore, magnetic property prediction studies of 2D magnetic materials can provide reliable references for magnetic property prediction studies of 2D semiconductor materials. The 2D magnetic materials mentioned in this paper include transition metal-based 2D materials with magnetic ordering characteristics and distorted van der Waals 2D magnetic materials, among others.

Some of these 2D magnetic materials also possess bandgap properties and can be classified as 2D magnetic semiconductors. To enhance the design efficiency of 2D magnetic materials with large magnetic moments and high magnetic anisotropy energy, the team led by Prasenjit Sen proposed a machine learning-based hierarchical screening strategy. This strategy first uses random forests and support vector machines to screen out materials with low thermodynamic stability, then classifies magnetic materials from non-magnetic materials, followed by regression prediction of magnetic moments for magnetic materials, and finally predicts and classifies materials with larger magnetic moments into two categories based on high/low magnetic anisotropy energy, thereby achieving hierarchical screening of materials. This hierarchical approach progressively narrows the range of candidate materials, significantly reducing the number of materials requiring first-principles calculations (DFT). Additionally, the study combines a random forest (RF) model to identify key features, reducing the number of features, thereby lowering computational costs and time. Specifically, if first-principles calculations were performed on all 278 materials, it would require approximately 13,900 core-hours of computation time. However, after ML screening, calculations were performed on only 13 candidate materials, resulting in an actual computation time of approximately 650 core-hours, a reduction of about 95%, effectively improving the efficiency of 2D magnetic material prediction [77]. This work highlights the potential of hierarchical machine learning strategies for efficient magnetic material screening. However, the framework still relies heavily on DFT-generated datasets and predefined descriptors.

In the field of magnetic Hamiltonian parameter identification and magnetic domain image generation research, Kyoung-Min Kim’s group introduced deep learning methods and developed two deep neural network models. One is used to estimate magnetic Hamiltonian parameters from magnetic domain images (regression model), and the other is used to generate magnetic domain images based on given magnetic parameters (generative model). The study first generated a dataset containing 17,292 paired images using atomic spin simulations, a computationally intensive method, and optimized image processing by simplifying it with fourfold rotational symmetry, thereby optimizing both data generation and preprocessing. These models are implemented through customized fully connected networks, enabling efficient and accurate analysis of the magnetic characteristics of distorted van der Waals magnets. Traditional atomic spin simulations require scanning a large number of parameters for a complete fit, resulting in high computational costs. However, using trained neural networks to analyze images can bypass the complex spin evolution process and directly extract features from the images, thereby reducing the demand for computational resources. Additionally, this deep learning method has the ability to automatically learn features and perform rapid inference, improving the efficiency of predicting magnetic Hamiltonian parameters and other properties [78]. Although this framework improves the efficiency of magnetic image analysis, its applicability to real experimental magnetic domains and complex magnetic environments still requires further validation.

Overall, machine learning can assist in magnetic parameter prediction and material screening in 2D magnetic material research. Research focused on efficiency improvements can reduce computational costs and experimental time, providing a feasible strategy for magnetic property prediction studies of 2D semiconductor materials with similar dimensions, structures, and properties.

##### Magnetic Property Prediction in 3D Magnetic Materials

Additionally, although three-dimensional magnetic materials (such as three-dimensional bulk magnetic materials) and 2D semiconductor materials exist in different dimensions, their research objectives for magnetic property prediction are the same: to identify and control the intrinsic magnetic parameters of materials. The magnetic mechanisms and data of three-dimensional materials can provide important prior knowledge and a foundation for transferring research findings to 2D semiconductor systems.

Research on three-dimensional magnetic materials can serve as a valuable reference for magnetic property prediction in 2D semiconductor materials. To enhance the prediction efficiency of magnetic performance and material stability, James R. Chelikowsky’s group developed a closed-loop feedback system integrating ML, DFT, adaptive genetic algorithms (AGA), and experimental validation. In this framework, a crystal graph convolutional neural network (CGCNN) was first employed to predict the formation energies of candidate structures, screening out thermodynamically stable materials with negative formation energies. Subsequently, DFT-based structural optimization and magnetic property prediction were performed on the filtered candidates, identifying rare-earth-free 3D magnetic compounds such as Fe_3_CoB_2_ with high magnetization and large magnetic anisotropy energy. Building upon these results, the researchers used the validated chemical compositions from the ML and DFT steps, namely various Fe-Co-B stoichiometries (such as Fe_3_CoB_2_ and FeCoB), as the search space for the AGA, enabling the discovery of lower-energy crystal structures. The predicted candidates were subsequently synthesized and experimentally verified, and the newly obtained low-energy structures and their properties were fed back into the ML model for retraining. This data iteration mechanism allowed the model to progressively adapt to the complex correlations between chemical composition and crystal structure, while its close coupling with experimental feedback reduced redundant computations and improved the efficiency of magnetic property prediction. However, the overall workflow remains computationally intricate, requiring the integration of multiple computational and experimental methodologies and depending strongly on the availability of high-quality initial datasets [79].

To predict magnetic Hamiltonian parameters, in 2020, a team from Xi’an Jiaotong University combined micromagnetic simulation and convolutional neural networks to propose a method for efficiently predicting the magnetic parameters of three-dimensional magnetic materials from experimental images. The framework employed simulation-based data augmentation strategies to construct large training datasets and optimized the neural network structure to directly predict continuous Hamiltonian parameters, thereby reducing computational complexity and improving prediction efficiency. Furthermore, the team proposed training the CNN using specific experimental images to directly extract corresponding magnetic parameters, thereby reducing computational time while maintaining reliable predictive accuracy. By employing efficient dataset generation and augmentation strategies, optimized model design, and improved experimental image adaptation methods, the framework effectively reduced the time and computational cost associated with traditional micromagnetic simulation fitting and related post-processing procedures. However, this method requires a large amount of simulated images as training data, and its generalization capability for experimental images still needs further validation [80].

In short, these two studies on three-dimensional magnetic materials provide valuable and transferable experience for the magnetic property prediction of 2D semiconductor materials. Both the closed-loop computational framework for efficient magnetic material screening proposed by James R. Chelikowsky’s team and the image-based magnetic parameter prediction method developed by the Xi’an Jiaotong University team can improve prediction efficiency and be extended to 2D systems, thus offering useful references for the magnetic characterization and application of 2D semiconductor materials. However, current studies still rely heavily on simulation-generated datasets and complex computational workflows, while the adaptability and reliability of these methods in practical two-dimensional semiconductor material systems still require further verification. Therefore, future research should further explore the reduction in computational complexity as well as deeper integration between machine learning prediction and experimental validation.

**Figure 6:**
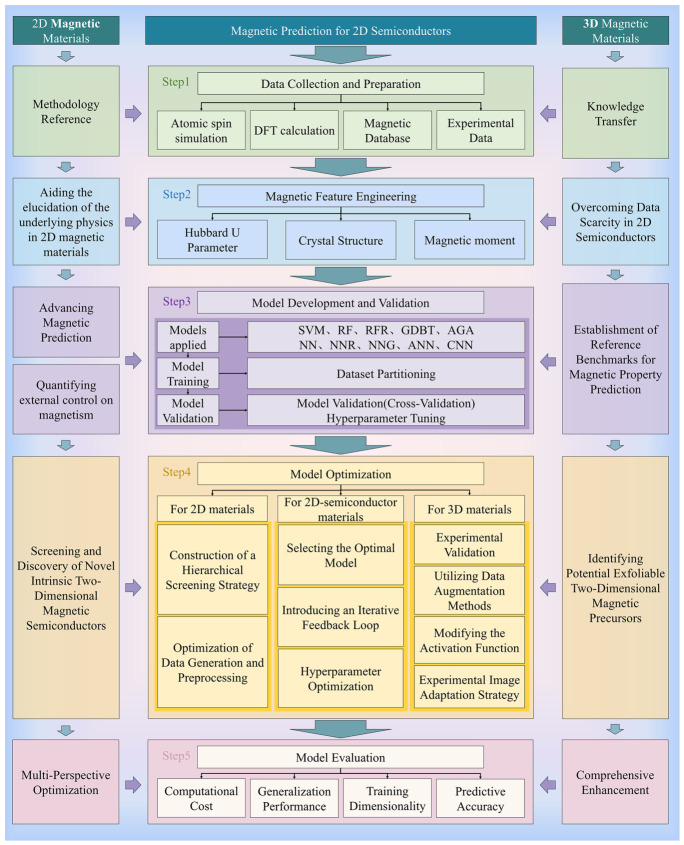
Key steps in magnetic property prediction for 2D semiconductor materials and methodological insights from magnetic property prediction in other materials.

Based on the reviewed literature, we summarize the main workflow of machine learning-based magnetic property prediction for 2D semiconductor materials, and we further summarize and analyze how magnetic property prediction in other material systems informs the study of 2D semiconductors, as illustrated in Figure 6. The figure highlights the key stages of the prediction pipeline, including feature engineering, model selection, model verification, optimization strategies, and model evaluation metrics, and it shows how studies on both two-dimensional and three-dimensional materials provide useful references at each of these stages, thereby supporting improvements in the accuracy of magnetic property prediction for 2D semiconductors. Among these stages, model selection and construction (Step 3) together with model optimization (Step 5) form the core of a high-accuracy prediction framework for 2D semiconductor magnetism. In Step 3, a multi-level and high-accuracy candidate model system can be established by combining existing methods for predicting the magnetism of 2D semiconductors with models that have been validated as effective in magnetic property prediction for both two-dimensional and three-dimensional materials. In Step 5, various optimization techniques, such as hierarchical screening, data augmentation, and improvements to activation functions and network architectures, can be jointly applied and iteratively refined based on experimental feedback, which is expected to further enhance the accuracy and robustness of the predictions. Beyond these core steps, fully incorporating experience from magnetic property prediction in other material systems can provide clear operational paradigms for 2D semiconductors in data acquisition, feature engineering, and model evaluation. By selectively transferring these cross-system approaches into different stages of the workflow, it becomes possible to alleviate the challenge of data scarcity for 2D materials, to accelerate the discovery of new intrinsic 2D magnetic semiconductors, and to establish a more reliable benchmark for magnetic property prediction in 2D semiconductor materials.

In summary, machine learning technology has made significant progress in the field of magnetic property prediction for 2D semiconductor materials. Its application in the prediction of magnetic properties for both two-dimensional and three-dimensional magnetic materials has provided important references for the former. Researchers have developed prediction models and frameworks by combining micromagnetic simulation, density functional theory, machine learning, and other advanced computational methods, thereby reducing computational costs and experimental time, improving data processing speed, and significantly enhancing the efficiency of magnetic property prediction for 2D semiconductor materials. However, current studies mainly focus on improving prediction efficiency and reducing computational cost, while some research frameworks still rely on relatively complex multi-step computational workflows and multi-model collaborative strategies, which to some extent increase the difficulty of practical application and large-scale implementation. In addition, although machine learning can significantly shorten the time required for magnetic property prediction, how to simultaneously maintain model stability and physical rationality while improving efficiency remains an important issue that requires further attention. Therefore, future research should further promote the simplification and optimization of efficient prediction frameworks and strengthen the integration between machine learning methods and practical material research demands.

#### 3.2.2. Research on Improving the Accuracy of Magnetic Property Predictions

High accuracy is of great significance for the prediction of magnetic properties for 2D semiconductor materials. The incorporation of machine learning facilitates more precise classification, thereby improving the accuracy of magnetic property predictions for 2D semiconductor materials. The predictive accuracy of the model not only directly influences the efficiency of candidate material screening but also determines the feasibility of subsequent experimental validation and device design. Therefore, how to improve the accuracy of prediction models under conditions of scarce data and complex features has become a key issue within this research field. To this end, a growing number of studies are focusing on enhancing the predictive capability of magnetic behavior in 2D semiconductor materials by introducing advanced machine learning algorithms, constructing physically constrained models, and integrating multiple material features.

The coefficient of determination R^2^ is one of the key indicators for evaluating the accuracy of magnetic property predictions and is used to measure how well a predictive model fits the data. The closer the value is to 1, the higher the accuracy. In 2023, Rhone’s team combined DFT and AI to develop a materials informatics framework, significantly improving the accuracy of predicting magnetic properties such as magnetic moment in transition metal halides and other materials. This method uses DFT calculations to provide precise labeled data on energy, magnetic moment, and magnetic excitation energy. It then employs semi-supervised learning to train neural networks using both labeled and large amounts of unlabeled data, overcoming the challenge of sparse labeled data. Specifically, the study incorporates magnetic excitation energy into the model’s loss function through physics-informed machine learning to better constrain the training process. Compared to a loss function that only includes the magnetic moment, this approach further reduces overfitting. Finally, by combining smooth overlap of atomic positions (SOAP) material descriptors with principal component analysis, multi-task learning, and other machine learning methods, the R^2^ value was improved from 0.2 to 0.8 [81]. In the same year, M. Modarresi’s team combined precise data generated by DFT with three machine learning algorithms (neural networks, random forests, and support vector regression) to successfully predict key physical properties such as Hubbard U parameters, unit cell parameters, and magnetic exchange parameters for transition metal chalcogenide/halide compounds. In the prediction of the three physical quantities, the R^2^ values for neural networks were 0.78, 0.88, and 0.95, respectively; for random forests, they were 0.72, 0.85, and 0.92, respectively; for support vector regression, they were 0.65, 0.80, and 0.88, respectively. Among them, the neural network demonstrated the highest accuracy owing to its multi-layer structure, excellent nonlinear fitting capability, and strong feature learning capability. However, the random forest and support vector regression had slightly lower accuracy than the neural network due to their weaker capacity to handle complex nonlinear relationships [75].

The reference [81] focuses on overcoming data resource constraints, enabling better utilization of unlabeled data to enhance model capabilities, but it also imposes higher requirements on data quality. Compared with the previous reference, the study [75] places greater emphasis on comparing the fitting performance of different machine learning methods. Although neural network models have higher complexity, they still offer the advantages of high accuracy.

In addition to using R^2^ as an indicator of model predictive accuracy, some groups also derive predictive accuracy using the following formula, which defines accuracy as the ratio of the number of correctly predicted samples to the total number of samples.

(1) Accuracy=Number of Correct Predictions / Total Number of Predictions

For example, in 2022, Acosta et al. employed an RF model to distinguish between magnetic and nonmagnetic materials across a dataset of 1713 compounds. The model achieved prediction accuracies of 84.5% for 474 magnetic materials and 95.9% for 1239 nonmagnetic materials, yielding an overall accuracy of 92.5%, which represents a weighted combination of both classes and reflects the proportion of correctly predicted samples across the entire dataset. Subsequently, the researchers applied the Sure Independence Screening and Sparsifying Operator (SISSO) to identify the most critical features and construct low-dimensional descriptors, achieving approximately 90% accuracy in predicting the magnetic order (ferromagnetic or antiferromagnetic) of magnetic materials. This approach demonstrated high-precision prediction of magnetism in 2D semiconductors by combining feature selection with interpretable machine learning algorithms [71]. The study integrated RF and SISSO algorithms, effectively leveraging atomic descriptors and crystal structural information to enhance the predictive accuracy of magnetic properties in 2D semiconductors. The combination of machine learning–assisted approaches has significantly improved the predictive accuracy of magnetic behaviors in 2D semiconductor systems and unveiled the richness and diversity of their magnetic phase diagrams.

In addition to R^2^ and the aforementioned formula, there are other metrics that can describe the accuracy of machine learning predictions of the magnetic properties of 2D semiconductor materials. For instance, in 2020, a research team from Xi’an Jiaotong University combined data augmentation techniques with micromagnetic simulations to generate synthetic datasets for training a convolutional neural network (CNN) based model. The model was directly applied to experimental magnetic domain images to estimate key magnetic parameters of soft magnetic materials. Taking the skyrmion radius as an example, a parameter that reflects the energy balance among the exchange energy, Dzyaloshinskii–Moriya interaction (DMI) energy, and demagnetization energy, the model yielded an estimated value of 24.28 nm, closely matching the experimental observation of 25.65 nm with a deviation of only 1.37 nm, thereby validating the model’s accuracy and reliability [80]. In 2022, Weibing Zhang and co-workers developed machine learning classifiers to predict and categorize the magnetic states of transition metal carbides and nitrides (MXenes), which include certain 2D semiconductor systems. They compared five algorithms, namely k-nearest neighbors (KNNs), random forest (RF), decision tree (DT), adaptive boosting (AdaBoost), and gradient boosting decision tree (GBDT). By incorporating the Coulomb matrix into a crystal-graph-based multilayer descriptor (CGMD) and applying average pooling together with elemental feature reduction, they constructed a compact yet efficient composite descriptor. After training with this optimized descriptor, the area under the receiver operating characteristic curve (AUC) values for all models improved from approximately 0.9 to 0.95, with the KNN model exhibiting a particularly notable increase from 0.815 to 0.945. This improvement was attributed to the inclusion of Coulomb interaction features, which enhanced model robustness, generalization capability, and overall predictive performance [82]. In 2023, Rhone et al. extracted a large dataset from density functional theory (DFT) calculations to train and optimize a random forest regression model. Using 55 descriptors as input features, they computed the reduction in mean squared error contributed by each descriptor to identify the most significant ones for predicting magnetic properties. This approach markedly improved the predictive accuracy for key parameters such as formation energy, magnetic moment, and bandgap in two-dimensional magnetic van der Waals materials. For instance, in the Mn_2_Bi_4_Te_8_ system, the predicted phonon dispersion exhibited no significant imaginary frequencies, confirming the dynamic stability of the material and indirectly validating the fidelity of the machine learning predictions [83]. More recently, in 2024, Kyoung-Min Kim and co-workers expanded the parameter space of twist angles and utilized atomistic spin simulations to construct a comprehensive, high-quality dataset. They further optimized a customized fully connected neural network through activation function selection and architectural refinement. This model achieved high-precision estimation of magnetic Hamiltonian parameters from domain images and accurate generation of domain images from given parameters. The model’s mean absolute percentage error (MAPE) was less than 4% for parameter estimation and 6% for image generation, demonstrating high predictive performance and strong robustness to noise and phase-boundary effects [78].

Collectively, these studies show that besides the commonly used R2 and the aforementioned formula, other metrics, including parameter deviation, AUC, phonon dispersion verification, and MAPE, effectively describe the predictive accuracy of 2D semiconductor magnetism, and combining physical methods with machine learning, along with these diverse metrics, lays a solid foundation for accurate prediction in low-dimensional materials. Nevertheless, current studies still lack unified evaluation standards across different datasets and prediction tasks, making direct comparisons between models difficult. In addition, although high predictive performance has been widely reported, the underlying physical mechanisms reflected by machine learning outputs remain insufficiently explored, which may limit the broader applicability and reliability of these models in more complex systems.

We have statistically summarized the literature covered in this section according to the materials, methods employed, and predictive accuracy metrics, as presented in Table 3. From this, we can briefly summarize as follows. Researchers have improved the generalization ability of models from multiple aspects, including the full utilization of data, the reasonable selection and optimization of models, and the construction and improvement of key descriptors, based on different machine learning methods and different types of predictive accuracy metrics, thereby improving the accuracy of magnetic property predictions for 2D semiconductor materials. In addition, different predictive tasks call for tailored evaluation metrics, and a multi-metric framework offers a more holistic assessment of model performance under diverse scenarios.

#### 3.2.3. Magnetic Property Prediction for Novel Material Discovery and Innovation

Magnetic property prediction methods combined with machine learning have been continuously innovated. In addition to making significant contributions to prediction efficiency and accuracy, they have also promoted the discovery and innovation of new materials. Machine learning, with its ability to efficiently process and analyze large amounts of material data, has enabled the rapid prediction of new material properties. Additionally, by identifying the complex relationships between material structure and properties, it provides guiding principles for new material design and accelerates the cycle from theoretical hypotheses to experimental validation. This section will summarize, in chronological order, the contributions made by various teams using different machine learning methods in magnetic property prediction research toward the discovery and innovation of new materials.

In 2020, Vivek B. Shenoy et al. employed a high-throughput computational and machine learning–integrated framework to systematically predict the magnetic ground states of 2D semiconductors, successfully identifying 18 novel candidates for magnetic topological materials, including ferromagnetic topological semimetals, axion insulators, and antiferromagnetic topological insulators. This pioneering study demonstrated the power of machine learning in large-scale material screening and discovery [84]. In 2022, Zhimei Sun’s group proposed an adaptive feedback optimization model that enabled the prediction and experimental validation of a novel rare-earth-free magnetic material, Fe_3_CoB_2_. Their work showed the potential of machine learning in small-data scenarios, providing a successful case study for few-shot learning in materials science [85]. In the same year, researchers from Dalian University of Technology developed a transition-metal-interconnected neural network (TMINN) for the rapid and accurate prediction of magnetic anisotropy energy (MAE) in 2D metal–organic frameworks (2D MOFs). Moreover, they proposed design principles based on W and Re elements, revealing that low coordination environments and oxygen-containing ligands (e.g., hydroxyl groups) favor the realization of enhanced MAE, thereby providing valuable theoretical guidance for the design of high-density magnetic storage materials [86]. In 2023, M. Modarresi et al. combined DFT with machine learning techniques to successfully identify ferromagnetic materials with high Curie temperatures and antiferromagnetic materials with high Néel temperatures, further illustrating the predictive power of data-driven models in magnetic materials research [75]. During the same year, Trevor David Rhone et al. developed an AI-based materials informatics framework that integrates high-throughput DFT computations with semi-supervised learning to overcome the challenge of label sparsity in materials datasets, thereby accelerating the discovery of stable van der Waals magnetic materials [81].

These studies share a consistent goal of accelerating the discovery of 2D magnetic materials by integrating machine learning with high-throughput or DFT computations. They gradually advance from large-scale screening to small-data learning, targeted property prediction, and label-sparse learning, collectively highlighting the key role of machine learning in magnetic material design and discovery.

#### 3.2.4. Magnetic Property Prediction for Research Repeatability and Transparency

The machine learning methods and models mentioned above not only contribute to predictive efficiency and accuracy, as well as the discovery and innovation of new materials, but also enhance the repeatability and transparency of research through standardized data processing and model training procedures. In this way, different research groups can obtain consistent results based on the same datasets and algorithms, thereby enhancing the reproducibility of their studies. For instance, in 2020, C. Won et al. estimated magnetic Hamiltonian parameters from experimentally observed images by employing a data-driven deep learning framework. They first generated synthetic magnetic domain images through Monte Carlo simulations by adding artificial noise with random amplitudes to mimic the complexity of experimental data. Subsequently, they constructed and trained three deep learning models, namely, ResNet18, ResNet50, and a customized convolutional neural network, to predict the Hamiltonian parameters. The accuracy of the models was then validated using SPLEEM experimental data. This standardized, data-driven workflow for data preprocessing and model training significantly improved the reproducibility of the research outcomes, facilitating validation and further innovation in subsequent experimental studies [87]. Similarly, Keisuke Takahashi et al. integrated data science with materials science to accelerate the discovery of magnetic 2D semiconductors. They first collected data for 216 2D materials and constructed a dataset using first-principles calculations. A Gaussian Naïve Bayes classifier was then applied to identify key descriptors and predict magnetic moments, enabling the generation of 746,496 possible material combinations. Through this approach, 254 2D materials with high magnetic moments were identified, among which eight novel stable magnetic materials were discovered [88]. These studies provided comprehensive details on model construction and training procedures and released raw datasets to the public, thereby promoting the reproducibility and transparency of machine learning-based materials prediction research.

Moreover, transparent model architectures and open-source codes allow for detailed examination and verification of research procedures and decision-making logic, further enhancing the transparency of scientific research. This openness not only promotes collaboration and trust within the scientific community but also accelerates the dissemination and application of innovative achievements. For example, in 2016, Sun et al. introduced residual learning and skip connections to construct models such as ResNet50, offering detailed insights into key module designs and effectively addressing optimization issues in deep neural networks [89]. In 2021, Hu et al. developed Swin Transformer, a novel hierarchical vision architecture that integrates shifted windows and achieves outstanding performance in tasks such as object detection. The authors also publicly released the source code and pretrained models, enabling independent verification and reuse by other researchers [90]. Building upon these two advances, in 2024, Zhang et al. proposed the Swin-ResNet50 model by replacing the 3 × 3 convolutional modules in the ResNet50 backbone with Swin Transformer blocks. This hybrid architecture effectively alleviates problems such as gradient explosion while improving computational efficiency in image processing. When applied to the magnetic property prediction of arsenic (As)-doped 2D semiconductors, the Swin-ResNet50 model, which was trained under identical conditions with the SGD optimizer, achieved an accuracy of 90.015%, outperforming both the original ResNet50 and Transformer-based models [91]. These studies demonstrate that by utilizing transparent architectures and open-source frameworks developed in previous work, researchers can quickly build and refine innovative models, substantially enhancing research efficiency and predictive accuracy. The open and transparent nature of machine learning promotes synergistic interactions among research results, facilitating mutual verification and complementary progress across different teams. Accordingly, this paradigm not only accelerates the advancement of magnetic property prediction in 2D semiconductors but also provides new perspectives and powerful tools for exploring complex physical systems.

In summary, recent advances in applying machine learning to magnetic property prediction in 2D semiconductor materials have demonstrated their significant advantages in terms of efficiency, accuracy, new material development, innovation, and research transparency. Machine learning models provide important theoretical guidance for new material design, performance optimization, and practical applications by automatically identifying the complex correlations between material structure and magnetic properties. Notably, machine learning excels in handling complex datasets and delivering high predictive accuracy. When integrated with traditional computational methods, it can further provide necessary physical constraints and causal interpretations, thereby ensuring the reliability and interpretability of prediction outcomes. However, machine learning still faces several challenges in predicting the magnetic properties of 2D semiconductor materials. For instance, the generalization ability and experimental data adaptability of such models require further validation, particularly when addressing small datasets and complex material systems [92]. In the future, further algorithm optimization, dataset expansion, and the introduction of additional physical constraints will facilitate the deep integration of machine learning and traditional computational methods, thereby continuously advancing the field of magnetic property prediction for 2D semiconductor materials.

### 3.3. Predictions of Other Physical Properties

In the field of performance prediction for 2D semiconductor materials, machine learning methods have been extensively employed not only for bandgap and magnetic property prediction, but also for the in-depth exploration of diverse properties such as thermal conductivity, optical characteristics, mechanical properties, carrier mobility, and chemical stability. These advances have substantially accelerated the application of 2D semiconductors in various domains, including electronic devices, energy conversion systems, and flexible electronics. Compared with conventional experimental approaches and computational methods, machine learning demonstrates remarkable advantages in terms of efficiency, accuracy, and cost-effectiveness.

#### 3.3.1. Thermal Conductivity

The thermal conductivity of 2D semiconductor materials, a key parameter for evaluating heat transport performance, plays a crucial role in the assessment of thermal management efficiency of electronic devices [93]. However, achieving high-accuracy predictions of thermal conductivity in 2D semiconductors remains a formidable challenge. Traditional first-principles approaches, such as those based on DFT combined with the Boltzmann transport equation (BTE), provide reliable accuracy but suffer from several inherent limitations. First, their computational cost becomes prohibitively high when dealing with large-scale or structurally complex systems, impeding their application to realistic device dimensions [94]. Second, for novel or heterogeneous structures, the lack of universal and transferable exchange–correlation functionals often results in significant discrepancies between simulated and experimental results [95]. Moreover, for heterostructures, thermal conductivity is governed by multiple coupled factors, including stacking configurations, grain boundaries, Moiré patterns, and lattice mismatch, which collectively complicate efficient and accurate prediction. These issues have constrained both the fundamental understanding and practical optimization of thermal transport in 2D semiconductors [96]. While these limitations of traditional first-principles methods are unlikely to be overcome in the near term, machine learning has emerged as a promising alternative for the prediction of thermal conductivity in 2D materials. For instance, in 2020, to improve predictive accuracy, Roche et al. integrated first-principles calculations, machine learning interatomic potentials (MLIPs) based on the moment tensor potential (MTP) framework, and molecular dynamics (MD) simulations to efficiently predict the thermal conductivity of monolayer C_3_N. The authors generated training data from phonon dispersion and MD simulations, constructed a high-precision interatomic potential using MTP, and performed non-equilibrium molecular dynamics (NEMD) simulations. Based on Fourier’s law under applied temperature gradients, they determined the intrinsic thermal conductivity of infinite-length C_3_N to be 418 ± 20 W m^−1^ K^−1^. This approach effectively combines the reproducibility of classical MD with the DFT-level accuracy of energy and force evaluations, thereby overcoming the overestimation of thermal conductivity often found in empirical potentials and the strong data dispersion of pure first-principles calculations [97]. In terms of computational efficiency, in 2024, Amon et al. further advanced ML-integrated modeling by coupling first-principles methods with MLIPs to predict the thermal conductivities of 2D semiconductors such as TiS_2_, MoS_2_, and their heterostructures. In their framework, short-range (intralayer) and long-range (interlayer) interactions were treated respectively using MTP and the D3 dispersion correction scheme. Through NEMD simulations, the team reported thermal conductivities of 15.40 W m^−1^ K^−1^ for bilayer TiS_2_, 11.41 W m^−1^ K^−1^ for bilayer MoS_2_, and 9.12 W m^−1^ K^−1^ for the TiS_2_/MoS_2_ heterostructure. By generating MLIPs from low-cost ab initio molecular dynamics (AIMD) trajectories, the study significantly reduced DFT computations and avoided redundant calculations, thereby enhancing computational efficiency and lowering research costs [96].

Collectively, these studies demonstrate that integrating machine learning with first-principles approaches provides a powerful and efficient route for high-fidelity thermal conductivity prediction, paving the way for the rational design of thermoelectric materials and heat management devices in next-generation nanoelectronics. Despite the promising progress of ML-assisted thermal conductivity prediction, current studies still face challenges in model generalization and physical interpretability, particularly for complex heterostructures and realistic device environments. Further integration of physics-based constraints and experimental validation will therefore be essential for improving the reliability and practical applicability of these approaches.

#### 3.3.2. Optical Properties

The optical properties of 2D semiconductor materials encompass metrics such as photoconversion efficiency and exciton binding energy [98]. These properties profoundly influence the materials’ application potential in photovoltaics and photodetectors. The optical properties of 2D semiconductor materials are closely linked to their bandgap characteristics. The bandgap directly determines properties such as the light absorption range, luminescence efficiency, and nonlinear optical response [99,100,101]. Consequently, once a model predicts the bandgap of a 2D semiconductor material, its optical properties can be further inferred based on the relationship between bandgap and optical characteristics. Thus, predicting the optical properties of 2D semiconductors often begins with predicting their bandgaps. Section 3.1 of this paper provides a comprehensive review of machine learning approaches for bandgap prediction in 2D semiconductors. This section focuses on introducing representative studies on optical property prediction using machine learning. For instance, in 2021, Hongjian Feng’s team combined machine learning with density functional theory (DFT) to predict the photovoltaic performance of 2D perovskites. They trained gradient boosting regressors (GBR), Extra Trees Regressors (ETR), and Random Forest Regressors (RF) on a dataset of 2303 perovskite materials. They then screened these materials based on bandgap predictions, identifying 83 compounds with suitable bandgaps (0.9–1.6 eV) to narrow the research scope. Subsequent stability testing revealed that Sr_2_VON_3_ and Ba_2_VON_3_ exhibited high theoretical power conversion efficiencies (PCE) of 30.35% and 26.03%, respectively. Furthermore, the study combined DFT simulations with ion implantation simulations for the ML-predicted material Sr_2_VON_3_ and revealed mechanisms, including non-adiabatic electron excitation, electron-phonon coupling, and electronic stopping power (ESP) triggered by low-energy ions traversing the material. These findings validated the optical tunability potential of ML-predicted materials, while maintaining physical plausibility, thereby providing new theoretical frameworks and strategies for designing high-performance optoelectronic devices [102]. This work demonstrates the potential of machine learning models in optical property prediction, establishing a methodological foundation for extending research to more complex optical characteristics such as absorption spectra and luminescence efficiency.

Overall, machine learning has shown considerable potential in accelerating the discovery and screening of optoelectronic materials by efficiently linking bandgap characteristics with optical performance. However, deeper modeling of complex optical mechanisms such as exciton behavior and light–matter interactions is lacking. Further improvement in model interpretability and experimental validation therefore remains necessary.

#### 3.3.3. Mechanical Properties

Mechanical properties are essential for assessing the structural stability, flexibility, and device applicability of 2D semiconductor materials. These include strength, hardness, toughness, Young’s modulus (elastic modulus), bending rigidity, and Poisson’s ratio [103], which together determine the performance and reliability of 2D semiconductor materials in practical applications [104]. Traditional computational approaches, such as DFT and molecular dynamics (MD) simulations, although widely employed, face inherent limitations in terms of computational efficiency, data correlation, model adaptability, and experimental compatibility [105]. In contrast, machine learning provides a data-driven framework that can efficiently capture complex, multi-parameter relationships, enabling inverse materials design and providing new opportunities to overcome these limitations. In terms of improving computational efficiency and predictive accuracy, in 2019, Wang et al. integrated MD simulations with ML techniques to investigate the mechanical behavior of monolayer WS_2_ systematically. Their study explored the effects of chirality, system size, temperature, strain rate, and random vacancy defects on key mechanical parameters, namely fracture strain, fracture strength, and Young’s modulus, while also revealing the mechanism of defect-induced stress concentration. Using an RF supervised learning algorithm trained on 3600 MD-generated datasets, they successfully established quantitative relationships between input features and target mechanical properties. The ML-predicted results exhibited excellent agreement with MD simulations, achieving mean squared errors (MSEs) of 9.2 × 10^−6^ for fracture strain, 1.9 × 10^−2^ GPa for fracture strength, and 3.8 GPa for Young’s modulus, all of which are several orders of magnitude smaller than the actual property values. This work demonstrated that ML can efficiently and accurately predict the mechanical performance of monolayer WS_2_, substantially reducing computational cost and time compared with conventional numerical simulations [106]. Regarding model selection for different application scenarios, in 2022, Islam et al. combined MD simulations with ML models to predict the mechanical properties of 2D transition-metal dichalcogenides (TMDs), including MoS_2_ and MoSe_2_. By using large-scale atomic/molecular massively parallel simulator (LAMMPS) to perform uniaxial tensile simulations on cracked TMD nanosheets, the authors constructed stress–strain datasets parameterized by chirality direction, temperature, and crack length, and trained two types of neural networks, a long short-term memory (LSTM) model and a feedforward neural network (FFNN) model. The LSTM network, which incorporated atomic mass and stress sequences as input, accurately reproduced the entire stress–strain curves with an R^2^ beyond 0.995, thus capturing the coupled effects of temperature and crack length on fracture behavior. The FFNN model, in contrast, directly predicted key mechanical quantities, including fracture stress, fracture strain, and Young’s modulus, with an R^2^ beyond 0.994. These results indicated that while the LSTM model is more capable of capturing dynamic mechanical responses, its higher computational complexity limits efficiency. Conversely, the FFNN provides rapid property estimation but cannot describe time-dependent deformation. This study thus established a multiscale ML–MD framework for predicting the mechanical behavior of 2D semiconductors, offering extensibility to other complex mechanical systems [107].

Collectively, these works underscore the growing potential of machine learning in accelerating the evaluation and optimization of mechanical properties in 2D semiconductors, providing essential insights for enhancing the fatigue life and mechanical reliability of flexible and nanoscale electronic devices. However, most current models are still trained on datasets derived from numerical simulations and remain limited in experimental validation. In addition, accurately describing long-term fatigue behavior, interfacial mechanics, and complex nonlinear deformation mechanisms in realistic device environments remains challenging for existing ML frameworks.

#### 3.3.4. Carrier Mobility

Carrier mobility, as a key parameter for evaluating charge transport efficiency in semiconductors, directly determines the switching speed of fabricated transistors. Consequently, researchers have long been devoted to the exploration and innovation of high-mobility materials. Traditional computational approaches require solving complex scattering integrals [108], with computational costs increasing exponentially with temperature, while machine learning is expected to effectively overcome such limitations. In 2024, a representative study on high-mobility material screening was reported by Qionghua Zhou et al. They developed a hybrid transfer learning framework that integrates adversarial transfer learning (ATL) with expert knowledge to identify promising target materials. Considering that bulk materials share certain chemical and physical similarities with 2D semiconductors and possess abundant theoretical and experimental datasets, the authors leveraged this information and further addressed the issue of data scarcity in 2D semiconductors. Through adversarial training, the model extracted shared representations between the two material systems, while expert knowledge was incorporated to characterize the unique structural and electronic features of 2D semiconductors, thereby enabling cross-material and cross-property knowledge transfer. By using crystal structure as the primary input, which fundamentally determines material properties, the model achieved rapid and accurate prediction of carrier mobility in 2D semiconductors, reaching a predictive accuracy exceeding 90% and a computational speed five orders of magnitude faster than conventional DFT calculations. Ultimately, the researchers screened 21 2D semiconductors with ultrahigh carrier mobility (>10^4^ cm^2^/V·s) and suitable bandgaps from 4266 candidate materials [109]. This study exemplifies how machine learning enables rapid and accurate prediction of carrier mobility in complex material systems, providing an efficient and reliable tool for high-mobility 2D semiconductor discovery and significantly accelerating the materials design process.

Overall, this work highlights the strong potential of machine learning, particularly transfer learning strategies, in accelerating the discovery of high-mobility 2D semiconductors while alleviating the problem of limited training data. The integration of expert knowledge with cross-material learning also provides an efficient route for screening large candidate material spaces. However, the predictive performance of current frameworks still depends heavily on the quality and compatibility of source-domain datasets, and their reliability under complex experimental conditions, such as impurity scattering and device-level transport environments, remains insufficiently verified.

#### 3.3.5. Chemical Stability

The chemical stability of 2D semiconductor materials not only affects structural integrity [110] but is also closely linked to mechanical properties [111], synthesis and processing technique [112], electronic structure [113], and long-term performance [114]. Existing studies have shown that certain 2D material systems, such as multilayer silicene, exhibit strong resistance to air degradation, further highlighting the importance of stability research for the practical applications of 2D materials [1]. Predicting chemical stability aids in screening materials with superior performance and extended service life, holding significant implications for material design, synthesis, application, and optimization of environmental adaptability [115]. Traditional methods (such as first-principles calculations) face limitations in predicting the stability of 2D semiconductor materials due to computational efficiency, empirical generalization, and dynamic environment simulation capabilities. However, the introduction of machine learning has driven new advancements in predicting the chemical stability of these materials. Stability prediction commonly employs two approaches: one directly predicts stability based on the material’s inherent atomic properties, while the other indirectly reflects stability by predicting formation energies [116]. A representative example of direct prediction was reported by Tundong Liu et al., who employed the C2DB database to extract 12 key physical descriptors, including atomic mass and density of states (DOS), and combined advanced feature engineering and data reduction strategies to predict the stability of 2D semiconductors. Five ML models, including RF, MLP, SVM, LR, and DT, were trained and compared, with the best model achieving an average accuracy of 0.9036. Furthermore, Shapley additive explanations (SHAP) and counterfactual analysis were employed to interpret feature importance, revealing that thermodynamic stability and Fermi energy are strongly and positively correlated with overall stability, while DOS at the Fermi level, total energy, number of atoms, and unit cell area exhibit negative correlations [117]. These findings demonstrate that ML can not only predict material stability with high accuracy but also provide quantitative insight into the relative contributions of distinct physical parameters, offering explicit guidance for materials screening and design. Notably, such direct-prediction methods require no detailed structural information, enabling rapid and large-scale screening of candidate materials. By contrast, the formation energy-based (indirect) approach is grounded in high-fidelity computational data, such as density functional theory (DFT) results, and is more suitable for fine-grained stability assessment and experimental validation of known structures. A notable example was reported by Schleder et al., who constructed a stability classification model using 3712 materials from the C2DB database. Their model integrated statistical features (atomic properties) and categorical features (material prototypes) to form a hybrid feature space, and employed an XGBoost classifier in combination with the Sure Independence Screening and Sparsifying Operator (SISSO) method for feature selection. The resulting model successfully categorized materials into low, medium, and high stability groups, achieving AUC values of 0.93, 0.89, and 0.94, respectively, demonstrating excellent classification capability. Moreover, the model predicted formation energies of high-stability materials with an average precision better than 0.2 eV/atom, quantitatively linking formation energy to stability. Specifically, materials with positive formation energy were classified as low stability, those with negative formation energy and an energy above the convex hull (ΔH_hull_) exceeding 0.2 eV/atom as medium stability, and those with negative formation energy and ΔH_hull_ below 0.2 eV/atom as high stability. Subsequent DFT calculations verified the ML predictions, showing strong consistency between computed and predicted results [118].

Overall, these studies confirm that formation energy prediction can effectively serve as an indirect indicator of chemical stability. Since stability arises from the interplay of multiple intrinsic properties, combining descriptors such as atomic attributes (e.g., Fermi level, DOS) and formation energy to construct accurate ML models not only enables efficient and reliable stability prediction but also elucidates the underlying interdependence among material characteristics, providing a solid foundation for mechanistic understanding and rational materials design.

In summary, machine learning has become a central tool in predicting diverse properties of 2D semiconductor materials, deeply permeating various branches of their performance research. Notably, the methodological frameworks employed in different prediction tasks exhibit both complementarity and a degree of universality. From a practical perspective, machine learning has made significant contributions to enhancing the accuracy and efficiency of property prediction. From a theoretical standpoint, it provides a solid foundation for uncovering the underlying mechanisms of materials science and advancing the development of interpretable theories.

## 4. Summary and Prospects

Predicting the properties of 2D semiconductor materials holds great significance, and the development and innovation of research methodologies have gradually become a focal point of attention. Machine learning is highly promising in addressing the limitations of traditional methods in terms of computational efficiency, data-processing capacity, and model generalization. The development of machine learning itself (such as the proposal of advanced algorithms) has greatly facilitated the property prediction of 2D semiconductor materials. Machine learning has been proven to improve the accuracy and efficiency of bandgap, magnetic, and other property predictions and to establish clear and reproducible research workflows, providing rapid and reliable experimental guidance for the design and screening of 2D semiconductors, thereby accelerating theoretical progress in the field of materials discovery.

Nevertheless, machine learning in the prediction of two-dimensional semiconductor material properties still faces numerous challenges. First, the data associated with two-dimensional semiconductor materials are highly diverse and originate from complex sources. Variations in computational parameters, exchange–correlation functionals, and experimental conditions adopted in different studies lead to a lack of consistency and standardization among datasets, thereby affecting the stability and reliability of model training. In addition, the insufficient availability of high-quality data and the imbalance of data distributions further limit the training performance and generalization capability of machine learning models. Second, the construction of machine learning features generally relies heavily on domain knowledge, and strong correlations or redundancies may exist among certain descriptors, thereby reducing training efficiency and prediction stability. Although dimensionality reduction techniques have been widely applied for feature optimization, current studies still insufficiently explore the complex coupling relationships between microscopic material structures and macroscopic properties. As a result, the physical interpretability of machine learning models remains limited, which to some extent restricts their further application in practical material design. Meanwhile, most current machine learning models are still trained and tested mainly on independently and identically distributed datasets, while their predictive capability across different databases, material systems, and unknown two-dimensional materials remains insufficiently validated. Although methods such as ensemble learning have improved model stability to a certain extent, achieving highly reliable cross-material-system prediction remains an important challenge for future research.

In light of these issues, this review summarizes several potential solutions reported in recent studies. First, automated data-processing pipelines integrated with natural language processing techniques could be developed to enable the automatic extraction of information from literature and databases. In addition, data augmentation, transfer learning, and multimodal data fusion methods may facilitate dataset expansion and cross-domain knowledge transfer, thereby improving data-processing efficiency and promoting the standardization of material datasets. This trend has already begun to emerge in recent studies [119]. Second, physical constraints may be incorporated into machine learning models to develop physics-informed machine learning approaches, thereby enhancing model interpretability and generalization capability. For example, graph neural networks (Graph Neural Networks, GNNs) can directly utilize crystal structure graph information to learn atomic interaction relationships, enabling more effective representation of the complex structure–property relationships within materials and facilitating the high-throughput screening and rapid discovery of two-dimensional magnetic materials [120,121]. Meanwhile, emerging techniques such as transfer learning, self-supervised learning, and large-scale pretrained models are also expected to improve the learning capability of machine learning models under small-sample conditions and enhance their cross-system prediction performance, as demonstrated in recent studies [122]. In addition, recent advances in agentic discovery frameworks may further accelerate the autonomous screening and optimization of two-dimensional semiconductor materials through intelligent workflow design and adaptive exploration of materials spaces [123,124].

The literature surveyed in this work indicates that standalone machine learning methods still face certain limitations in terms of interpretability and generalization. Consequently, one of the most promising future research directions may lie in the synergistic integration of machine learning with traditional computational approaches and experimental techniques. ML can accelerate preliminary screening of material properties and provide effective support in cases where experimental data are scarce or computational costs are prohibitive. In turn, traditional methods and experimental techniques supply ML with high-quality training and validation data, while also offering a solid theoretical foundation. Therefore, we infer that the future of property prediction in 2D semiconductor materials lies in the deep integration of machine learning with traditional and experimental methods. Such synergistic integration can fully leverage the respective strengths of both sides, endowing such research with not only high efficiency and strong predictive capability but also rigor, reliability, and physical interpretability. This combination will further promote the development and applications of machine learning in the research of 2D semiconductor materials.

## Figures and Tables

**Table 1:** Overview of the organization and key contents of this review.

Section	Main Topic	Organization Logic and Key Content
Section 1	Introduction	Introduces 2D semiconductor research background and importance of ML-assisted property prediction.
Section 2	Fundamental Properties and Prediction Strategies	Reviews key properties, compares conventional computational methods with ML, and highlights advantages of data-driven approaches.
Section 3	Research progress of machine learning predicting properties	Summarizes the research progress of machine learning in predicting bandgap, magnetic, and other properties of 2D semiconductor materials using different organizational frameworks.
Section 3.1	Bandgap Prediction	Organized by ML model type: single algorithms, ensemble models, and neural networks.
Section 3.2	Magnetic Property Prediction	Organized by ML advantages: efficiency, accuracy, material discovery, and reproducibility.
Section 3.3	Prediction of Other Physical Properties	Summarizes prediction of chemical stability, carrier mobility, and other relevant properties.
Section 4	Summary and Prospects	Discusses challenges, future research directions including database construction, descriptor optimization, and multimodal learning strategies.

**Table 2:** Comparison of traditional vs. machine learning methods for property prediction of 2D semiconductor materials.

Aspect	Traditional Methods	Machine Learning
Representative methods	DFT, MD, Monte Carlo simulations…	SVM, RF, CNN,…
Computational efficiency	Low efficiency; high computational cost	High efficiency after training
Physical interpretability	Strong physical basis	Limited interpretability
Prediction accuracy	Reliable but system-dependent	High with sufficient data
Generalization capability	Limited for unexplored systems	Potentially strong but data-dependent
Autonomous learning ability	Absent	data-driven feature learning

**Table 3:** Performance comparison of various machine learning methods for predicting magnetic properties of 2D semiconductors.

Materials	Methods	Predictive Accuracy Metrics (Note: “——” in the Table Indicates That the Metric Was Not Reported in the Original Source.)						
		R^2^					Formula-Derived Accuracy Values	Other Metrics (Interpolation, AUC, MSE, and MAPE)
Transition Metal Halides [81]	DFT + AI	0.8					——	——
Transition Metal Chalcogenides/Halides and Related Compounds [75]	Neural Network RF SVR		Parameters	Hubbard U	Lattice Params	MEPs	——	——
		Methods						
		Neural Network		0.78	0.88	0.95		
		RF		0.72	0.85	0.92		
		SVR		0.65	0.80	0.88		
2D Materials [71]	RF	——					92.5%	——
FeGe, FeGe_0.5_Si_0.5_, etc. [80]	CNN Sliding Window Data Augmentation Sigmoid Output Layer	——					——	∆ = 1.37 nm
2D MXene Materials [82]	KNN RF DT AdaB GBDT	——					——	AUC = 0.95
2D Van der Waals Magnetic Materials [83]	RFR	——					——	MSE decline
Twisted 2D Van der Waals Magnetic Materials [78]	FNN	——					——	Parameter Estimation: MAPE < 4% Image Generation: MAPE < 6%

## Data Availability

No new data were created or analyzed in this study.

## Abbreviations

The following abbreviations are used in this manuscript:

DFT	Density functional theory
2D semiconductor	Two-dimensional semiconductor
MAPE	Mean absolute percentage error
MD	Molecular dynamics
ML	Machine learning
RMSE	Root mean square error
